# Supplementary material for: Changes in anxiety and depression levels and meat intake following recognition of low genetic risk for high body mass index, triglycerides, and lipoproteins: A randomized controlled trial
Source: PLoS One. 2023 Sep 8;18(9):e0291052. doi: 10.1371/journal.pone.0291052 (PMC10490956; doi:10.1371/journal.pone.0291052)
Supplement: S1 Table — (DOCX) [file pone.0291052.s002.docx]

**S1 Table. Overview of the DTC test results provided to the study subjects.**

| **Test Item** | **Gene** | **SNP** | **Overall results** | **Descriptions on possessing risk allele** | **Recommendation for possessing risk allele** |
| --- | --- | --- | --- | --- | --- |
| **Body Fat Mass** | FTO | rs9939609 | Good / Borderline risk / Caution | There is a high risk of obesity by excessively storing the remaining calories in fat. | Low-fat meals are recommended. |
|  | MC4R | rs17782313 |  | There is a high risk of obesity by increasing appetite and eating snacks frequently. | It is recommended to reduce snack intake. |
|  | BDNF | rs6265 |  | Social stress can increase appetite as a reward. | Be careful of overeating or binge eating even in stressful or depressive situations. |
| **Triglyceride** | GCKR | rs780094 | Good / Borderline risk / Caution | Triglyceride production increases. | It is recommended to consume nuts or green tea that can lower triglyceride levels. |
|  | ANGPTL3 | rs10889353 |  | The amount of neutral fat in the blood increases. |  |
|  | MLXIPL | rs17145738 |  | The amount of fat accumulated in the liver increases. |  |
|  | TRIB1 | rs2954029 |  | It secretes a lot of fat from the liver to the blood. |  |
| **LDL-Cholesterol** | SORT1 | rs646776 | Good / Borderline risk / Caution | The amount of LDL cholesterol in the blood is relatively high. | It is recommended to eat less cholesterol-rich foods. |
|  | HMGCR | rs12654264 |  | The blood cholesterol concentration is high due to the increase in the rate of cholesterol production in the liver. |  |
|  | ABO | rs635634 |  | The decomposition rate of LDL cholesterol in the blood decreases. |  |
| **HDL-Cholesterol** | ABCA1 | rs1883025 | Good / Borderline risk / Caution | It reduces the emission of HDL cholesterol from cells to cells. | Aerobic exercise, weight loss, smoking cessation, and eating fruit and vegetables are recommended to increase HDL cholesterol levels. |
|  | MYL2 | rs12229654 |  | It reduces the amount of HDL cholesterol and increases the risk of metabolic syndrome in Koreans. |  |
|  | LIPG | rs4939883 |  | The amount of HDL cholesterol in the blood decreases. |  |
|  | CETP | rs1532624 |  |  |  |
