# Supplementary material for: Changes in anxiety and depression levels and meat intake following recognition of low genetic risk for high body mass index, triglycerides, and lipoproteins: A randomized controlled trial
Source: PLoS One. 2023 Sep 8;18(9):e0291052. doi: 10.1371/journal.pone.0291052 (PMC10490956; doi:10.1371/journal.pone.0291052)
Supplement: S2 Table — (DOCX) [file pone.0291052.s003.docx]

**S2 Table. Classification of foods into 23 food groups.**

| No | Categories | No | 23 Food groups |
| --- | --- | --- | --- |
| 1 | Grains | 1 | Grains |
|  |  |  |  |
|  |  | 2 | Noodles |
|  |  | 3 | Ramen |
|  |  | 4 | Bread |
|  |  | 5 | Cereals |
|  |  | 6 | Potato and starches |
|  |  | 7 | Snacks |
|  |  | 8 | Sugar |
| 2 | Meat/Fish/Eggs/Poultry/Beans | 9 | Meat |
|  |  | 10 | Processed meat |
|  |  | 11 | Fish and shells |
|  |  | 12 | Eggs |
|  |  | 13 | Beans |
| 3 | Vegetables | 14 | Vegetables |
|  |  | 15 | Seaweeds |
|  |  | 16 | Kimchi |
| 4 | Fruits | 17 | Fruits |
|  |  | 18 | Fruit juice |
| 5 | Dairy product | 19 | Dairy |
| 6 | Fat or oil | 20 | Fat or oil |
|  |  |  |  |
| 7 | Beverage or fast food | 21 | Sugar-sweetened beverages (SSB) |
|  |  | 22 | Beverages without sugar |
|  |  | 23 | Fast foods |
