# Supplementary material for: Changes in anxiety and depression levels and meat intake following recognition of low genetic risk for high body mass index, triglycerides, and lipoproteins: A randomized controlled trial
Source: PLoS One. 2023 Sep 8;18(9):e0291052. doi: 10.1371/journal.pone.0291052 (PMC10490956; doi:10.1371/journal.pone.0291052)
Supplement: S3 Table — (DOCX) [file pone.0291052.s004.docx]

**S3 Table. Generalized anxiety disorder-7 (GAD-7) and Patient Health Questionnaire-9 (PHQ-9).**

| **Generalized Anxiety Disorder-7 (GAD-7)** | | | | | |
| --- | --- | --- | --- | --- | --- |
| Over the last 2 weeks, how often have you  been bothered by the following problems? (Use “✔” to indicate your answer) | | Not at all | Several days | More than half the days | Nearly every day |
| 1 | Feeling nervous, anxious or on edge | 0 | 1 | 2 | 3 |
| 2 | Not being able to stop or control worrying | 0 | 1 | 2 | 3 |
| 3 | Worrying too much about different things | 0 | 1 | 2 | 3 |
| 4 | Trouble relaxing | 0 | 1 | 2 | 3 |
| 5 | Being so restless that it is hard to sit still | 0 | 1 | 2 | 3 |
| 6 | Becoming easily annoyed or irritable | 0 | 1 | 2 | 3 |
| 7 | Feeling afraid as if something awful might happen | 0 | 1 | 2 | 3 |
| Reference: http://www.phqscreeners.com/select-screener | | | | | |
| **Patient Health Questionnaire-9 (PHQ-9)** | | | | | |
| Over the last 2 weeks, how often have you been bothered by any of the following problems? (Use “✔” to indicate your answer) | | Not at all | Several days | More than half the days | Nearly every day |
| 1 | Little interest or pleasure in doing things | 0 | 1 | 2 | 3 |
| 2 | Feeling down, depressed, or hopeless | 0 | 1 | 2 | 3 |
| 3 | Trouble falling or staying asleep, or sleeping too much | 0 | 1 | 2 | 3 |
| 4 | Feeling tired or having little energy | 0 | 1 | 2 | 3 |
| 5 | Poor appetite or overeating | 0 | 1 | 2 | 3 |
| 6 | Feeling bad about yourself — or that you are a failure or have let yourself or your family down | 0 | 1 | 2 | 3 |
| 7 | Trouble concentrating on things, such as reading the newspaper or watching television | 0 | 1 | 2 | 3 |
| 8 | Moving or speaking so slowly that other people could have noticed? Or the opposite — being so fidgety or restless that you have been moving around a lot more than usual | 0 | 1 | 2 | 3 |
| 9 | Thoughts that you would be better off dead or of hurting yourself in some way | 0 | 1 | 2 | 3 |
|  | | Not difficult at all | Somewhat difficult | Very difficult | Extremely difficult |
| If you checked off any problems, how difficult have these problems made it for you to do your work, take care of things at home, or get along with other people? | | 0 | 1 | 2 | 3 |
| Reference: http://www.phqscreeners.com/select-screener | | | | | |
