# Supplementary material for: Changes in anxiety and depression levels and meat intake following recognition of low genetic risk for high body mass index, triglycerides, and lipoproteins: A randomized controlled trial
Source: PLoS One. 2023 Sep 8;18(9):e0291052. doi: 10.1371/journal.pone.0291052 (PMC10490956; doi:10.1371/journal.pone.0291052)
Supplement: S4 Table — (DOCX) [file pone.0291052.s005.docx]

**S4 Table. International physical activity questionnaire (IPAQ)-short form**

| **International physical activity questionnaire (IPAQ)-short form** | |
| --- | --- |
| **The questions will ask you about the time you spent being physically active in the last 7 days. Please answer each question even if you do not consider yourself to be an active person. Please think about the activities you do at work, as part of your house and yard work, to get from place to place, and in your spare time for recreation, exercise or sport.** | |
| Think about all the vigorous activities that you did in the last 7 days. Vigorous physical activities refer to activities that take hard physical effort and make you breathe much harder than normal. Think only about those physical activities that you did for at least 10 minutes at a time. | |
| 1. | During the last 7 days, on how many days did you do vigorous physical activities like heavy lifting, digging, aerobics, or fast bicycling? |
|  | _____ days per week   - No vigorous physical activities → Skip to question 3 |
| 2. | How much time did you usually spend doing vigorous physical activities on one of those days? |
|  | _____ hours per day _____ minutes per day   - Don’t know/Not sure |
| Think about all the moderate activities that you did in the last 7 days. Moderate activities refer to activities that take moderate physical effort and make you breathe somewhat harder than normal. Think only about those physical activities that you did for at least 10 minutes at a time. | |
| 3. | During the last 7 days, on how many days did you do moderate physical  activities like carrying light loads, bicycling at a regular pace, or doubles tennis? Do not include walking. |
|  | _____ days per week   - No moderate physical activities → Skip to question 5 |
| 4. | How much time did you usually spend doing moderate physical activities on one of those days? |
|  | _____ hours per day _____ minutes per day  Don’t know/Not sure |
| Think about the time you spent walking in the last 7 days. This includes at work and at home, walking to travel from place to place, and any other walking that you have done solely for recreation, sport, exercise, or leisure. | |
| 5. | During the last 7 days, on how many days did you walk for at least 10 minutes  at a time? |
|  | _____ days per week   - No walking → Skip to question 7 |
| 6. | How much time did you usually spend walking on one of those days? |
|  | _____ hours per day  _____ minutes per day  Don’t know/Not sure |
| The last question is about the time you spent sitting on weekdays during the last 7 days. Include time spent at work, at home, while doing course work and during leisure time. This may include time spent sitting at a desk, visiting friends, reading, or sitting or lying down to watch television. | |
| 7. | During the last 7 days, how much time did you spend sitting on a week day? |
|  | _____ hours per day _____ minutes per day   - Don’t know/Not sure |
