# Supplementary material for: Changes in anxiety and depression levels and meat intake following recognition of low genetic risk for high body mass index, triglycerides, and lipoproteins: A randomized controlled trial
Source: PLoS One. 2023 Sep 8;18(9):e0291052. doi: 10.1371/journal.pone.0291052 (PMC10490956; doi:10.1371/journal.pone.0291052)
Supplement: S5 Table — 1) Change = food intake at follow-up–food intake at baseline. 2) Means with different superscripts indicate the significant differences in changes in food intakes among CON, INR, and IR groups by one-way ANOVA tests and Kruskal-Wallis tests, followed by Bonferroni-correction multiple comparison tests. The asterisks indicate a significant difference (*: P value < 0.05) compared to food intakes from the baseline to the follow-up time point. c Added sugar includes sugar, fruit juice, and sugar-sweetened beverages. CON, control; ILR, Intervention-Low Risk; Intervention-High Risk. (DOCX) [file pone.0291052.s006.docx]

**S5 Table. Differences in food intakes among CON, ILR, and IHR groups ^1), 2), 3)^**

| **Male subjects (*n* = 50)** | | | | | | | |
| --- | --- | --- | --- | --- | --- | --- | --- |
| **Parameter** | **CON**  **(*n* = 17)** | | **ILR**  **(*n* = 16)** | | **IHR**  **(*n* = 17)** | | ***P* value ^2)^** |
|  | **Mean (SEM)** | **Change ^1)^** | **Mean (SEM)** | **Change ^1)^** | **Mean (SEM)** | **Change ^1)^** |  |
| **Meat (g/d)** | | | | | | | |
| Baseline | 132.7 (20.1) |  | 107.6 (19.2) |  | 162.4 (18.7) |  |  |
| 3-month follow-up | 127.4 (21.2) | -5.3 (30.3) | 157.0 (27.8) | 49.4 (30.8) | 110.2 (14.2) | -52.2 (25.4) | 0.056 |
| 6-month follow-up | 141.5 (19.3) | 8.8 (30.1) | 128.1 (25.6) | 20.4 (31.1) | 165.0 (21.9) | 2.6 (19.3) | 0.989 |
| **Processed meat (g/d)** | | | | | | | |
| Baseline | 15.5 (3.4) |  | 19.1 (5.4) |  | 13.5 (3.3) |  |  |
| 3-month follow-up | 13.2 (3.9) | 1.6 (4.0) | 13.6 (3.1) | -4.9 (7.2) | 17.7 (5.9) | 2.2 (9.4) | 0.823 |
| 6-month follow-up | 15.7 (4.0) | 4.1 (4.4) | 10.7 (4.5) | -7.7 (8.3) | 15.3 (5.5) | -0.3 (6.2) | 0.432 |
| **Fruits (g/d)** | | | | | | | |
| Baseline | 71.0 (24.5) |  | 32.5 (11.2) |  | 133.0 (32.7) |  |  |
| 3-month follow-up | 39.9 (13.5) | -31.0 (19.3) ^ab^ | 79.6 (30.9) | 47.1 (31.3) ^a^ | 50.4 (18.2) **^*^** | -82.6 (32.5) ^b^ | 0.044 |
| 6-month follow-up | 76.4 (16.5) | 5.4 (23.9) | 28.9 (8.1) | -3.6 (12.1) | 53.2 (18.3) | -79.8 (39.8) | 0.249 |
| **Added sugar (g/d)** | | | | | | | |
| Baseline | 140.2 (44.6) |  | 61.2 (16.7) |  | 86.5 (25.8) |  |  |
| 3-month follow-up | 77.6 (17.8) | -62.6 (51.3) | 89.4 (23.5) | 28.2 (28.7) | 113.8 (35.7) | 27.3 (50.2) | 0.373 |
| 6-month follow-up | 83.1 (33.6) | -57.1 (48.9) ^b^ | 84.8 (20.3) | 23.6 (25.6) ^ab^ | 165.9 (41.0) **^*^** | 79.4 (37.1) ^a^ | 0.036 |
| **Female subjects (*n* = 50)** | | | | | | | |
| **Parameter** | **CON**  **(*n* = 18)** | | **ILR**  **(*n* = 16)** | | **IHR**  **(*n* = 16)** | | ***P* value ^2)^** |
|  | **Mean (SEM)** | **Change ^1)^** | **Mean (SEM)** | **Change ^1)^** | **Mean (SEM)** | **Change ^1)^** |  |
| **Meat (g/d)** | | | | | | | |
| Baseline | 76.6 (11.3) |  | 78.6 (13.3) |  | 116.6 (25.7) |  |  |
| 3-month follow-up | 77.3 (16.3) | 0.6 (19.3) | 112.2 (18.0) | 33.6 (23.9) | 100.5 (14.3) | -16.2 (29.7) | 0.657 |
| 6-month follow-up | 89.1 (17.9) | 12.5 (18.5) | 75.4 (21.5) | -3.2 (24.4) | 93.0 (16.7) | -23.6 (29.9) | 0.919 |
| **Processed meat (g/d)** | | | | | | | |
| Baseline | 10.8 (2.6) |  | 13.4 (3.5) |  | 7.1 (2.4) |  |  |
| 3-month follow-up | 11.0 (2.4) | 0.2 (3.3) | 12.3 (5.2) | -1.2 (6.2) | 18.2 (5.8) | 11.1 (6.1) | 0.190 |
| 6-month follow-up | 9.9 (3.6) | -0.9 (4.1) | 10.8 (2.9) | -2.6 (4.5) | 11.7 (5.1) | 4.6 (5.5) | 0.952 |
| **Fruits (g/d)** | | | | | | | |
| Baseline | 96.6 (19.0) |  | 115.8 (28.5) |  | 90.1 (18.7) |  |  |
| 3-month follow-up | 95.7 (25.1) | -0.9 (29.4) | 83.4 (31.9) **^*^** | -32.4 (39.7) | 47.0 (9.6) | -43.1 (16.9) | 0.451 |
| 6-month follow-up | 60.9 (15.0) | -35.7 (23.7) | 92.0 (27.9) | -23.9 (23.7) | 83.6 (35.0) | -6.6 (28.4) | 0.906 |
| **Added sugar (g/d)** | | | | | | | |
| Baseline | 104.3 (21.6) |  | 62.3 (23.4) |  | 37.1 (12.4) |  |  |
| 3-month follow-up | 62.5 (22.1) | -41.8 (21.4) | 47.0 (14.9) | -15.3 (24.5) | 41.8 (11.7) | 4.6 (10.7) | 0.277 |
| 6-month follow-up | 59.9 (12.8) ^*^ | -44.4 (20.8) | 70.4 (23.1) | 8.1 (33.6) | 59.8 (20.7) | 22.7 (16.5) | 0.206 |

**^1)^** Change = food intake at follow-up – food intake at baseline.

**^2)^** Means with different superscripts indicate the significant differences in changes in food intakes among CON, INR, and IR groups by one-way ANOVA tests and Kruskal-Wallis tests, followed by Bonferroni-correction multiple comparison tests. The asterisks indicate a significant difference (*: P value < 0.05) in food intakes from the baseline to the follow-up time point.

**^3)^** Added sugar includes sugar, fruit juice, and sugar-sweetened beverages.

CON, control; ILR, Intervention-Low Risk; Intervention-High Risk
