# Supplementary material for: Changes in anxiety and depression levels and meat intake following recognition of low genetic risk for high body mass index, triglycerides, and lipoproteins: A randomized controlled trial
Source: PLoS One. 2023 Sep 8;18(9):e0291052. doi: 10.1371/journal.pone.0291052 (PMC10490956; doi:10.1371/journal.pone.0291052)
Supplement: S6 Table — 1) Change = food intake at follow-up–food intake at baseline. 2) Means with different superscripts indicate the significant differences in changes in PHQ-9 and GAD-7 scores or categories among CON, INR, and IR groups by one-way ANOVA tests and Kruskal-Wallis tests, followed by Bonferroni-correction multiple comparison tests. The asterisks indicate a significant difference (*: P value < 0.05) compared to PHQ-9 or GAD-7 from the baseline to the follow-up time point. CON, control; ILR, Intervention-Low Risk; Intervention-High Risk. (DOCX) [file pone.0291052.s007.docx]

**S6 Table. Differences in anxiety and depression levels among CON, ILR, and IHR groups ^1), 2)^**

| **Parameter** | **CON**  **(*n* = 35)** | | **ILR**  **(*n* = 32)** | | **IHR**  **(*n* = 33)** | | ***P* value ^2)^** |
| --- | --- | --- | --- | --- | --- | --- | --- |
|  | **Mean (SEM)** | **Change ^1)^** | **Mean (SEM)** | **Change ^1)^** | **Mean (SEM)** | **Change ^1)^** |  |
| **GAD-7_Score** | | | | | | | |
| Baseline | 3.2 (0.6) |  | 4.3 (0.8) |  | 3.5 (0.5) |  |  |
| 3-month follow-up | 3.5 (0.7) | 0.31 (0.64) | 3.6 (0.8) | -0.78 (0.84) | 3.5 (0.5) | 0.03 (0.67) | 0.862 |
| 6-month follow-up | 3.8 (0.8) | 0.57 (0.80) | 3.4 (0.5) | -1.13 (0.73) | 3.4 (0.5) | -0.09 (0.62) | 0.993 |
| **PHQ-9_Score** | | | | | | | |
| Baseline | 4.5 (0.8) |  | 6.8 (0.9) |  | 4.2 (0.5) |  |  |
| 3-month follow-up | 5.0 (0.8) | 0.46 (0.70) | 5.4 (0.9) | -1.31 (0.80) | 4.7 (0.6) | 0.45 (0.67) | 0.241 |
| 6-month follow-up | 5.4 (1.0) | 0.91 (1.06) ^a^ | 4.2 (0.7) ***** | -2.53 (0.78) ^b^ | 4.4 (0.6) | 0.21 (0.62) ^ab^ | 0.022 |

**^1)^** Change = GAD-7 or PHQ-9 score at follow-up – GAD-7 or PHQ-9 score at baseline.

**^2)^** Means with different superscripts indicate the significant differences in changes in PHQ-9 and GAD-7 scores or categories among CON, INR, and IR groups by one-way ANOVA tests and Kruskal-Wallis tests, followed by Bonferroni-correction multiple comparison tests.

The asterisks indicate a significant difference (*: P value < 0.05) in the PHQ-9 or GAD-7 score from the baseline to the follow-up time point.

CON, control; ILR, Intervention-Low Risk; Intervention-High Risk
