# Supplementary material for: Changes in anxiety and depression levels and meat intake following recognition of low genetic risk for high body mass index, triglycerides, and lipoproteins: A randomized controlled trial
Source: PLoS One. 2023 Sep 8;18(9):e0291052. doi: 10.1371/journal.pone.0291052 (PMC10490956; doi:10.1371/journal.pone.0291052)
Supplement: S7 Table — *Significant at P<0.001. r = Spearman’s correlation coefficient. (DOCX) [file pone.0291052.s008.docx]

**S7 Table. Spearman’s correlation between meat intake and nutrients intake**

| Variables | Total (*n* = 100) | |
| --- | --- | --- |
|  | ***r*** | ***P* value** |
| Protein (% energy/d) | 0.437 * | <0.001 |
| Thiamin (mg/d) | 0.310 * | <0.001 |
| Vitamin B6 (mg/d) | 0.397 * | <0.001 |

* Significant at *P*<0.001. r= Spearman’s correlation coefficient
