# Supplementary material for: Changes in anxiety and depression levels and meat intake following recognition of low genetic risk for high body mass index, triglycerides, and lipoproteins: A randomized controlled trial
Source: PLoS One. 2023 Sep 8;18(9):e0291052. doi: 10.1371/journal.pone.0291052 (PMC10490956; doi:10.1371/journal.pone.0291052)
Supplement: S8 Table — 1) MET: metabolic equivalent task. 2) The R2 values for GAD-7 scores at 3-month and 6-month were 0.072 and 0.024, respectively. The R2 values for PHQ-9 scores at 3-month and 6-month were 0.050 and 0.011, respectively. ILR, Intervention-Low Risk; BMI, body mass index; PA, physical activity; GAD-7, Generalized Anxiety Disorder 7-item scale; PHQ-9, Patient Health Questionnaire 9-item scale. (DOCX) [file pone.0291052.s009.docx]

**S8 Table. Multiple linear regression analysis on the association between GAD-7 or PHQ-9 scores and meat intake in total subjects ^1), 2)^**

|  | **Total (n=100)** | | | | | | | | | | | |
| --- | --- | --- | --- | --- | --- | --- | --- | --- | --- | --- | --- | --- |
|  | **GAD-7 score at 3-month** | | | **GAD-7 score at 6-month** | | | **PHQ-9 score at 3-month** | | | **PHQ-9 score at 6-month** | | |
|  | **B (SE)** | **Standardized *β*** | ***p*** | **B (SE)** | **Standardized *β*** | ***p*** | **B (SE)** | **Standardized *β*** | ***p*** | **B (SE)** | **Standardized *β*** | ***p*** |
| **Meat intake (g/d)** | -0.002 (0.005) | -0.042 | 0.683 | -0.000 (0.005) | -0.002 | 0.986 | -0.002  (0.006) | -0.043 | 0.681 | -0.002  (0.006) | -0.039 | 0.726 |
| **Women vs. men** | 2.391 (1.061) | 0.303 | 0.027 | -0.277 (1.076) | -0.036 | 0.797 | 2.332  (1.220) | 0.260 | 0.059 | -0.410  (1.277) | -0.045 | 0.749 |
| **Age (years)** | 0.134 (0.190) | 0.072 | 0.482 | -0.145 (0.191) | -0.080 | 0.451 | 0.054  (0.218) | 0.026 | 0.804 | -0.189  (0.227) | -0.088 | 0.407 |
| **BMI (kg/m^2^)** | 0.127 (0.256) | 0.064 | 0.622 | -0.000 (0.258) | -0.146 | 0.276 | 0.244  (0.294) | 0.109 | 0.409 | -0.007  (0.306) | -0.003 | 0.983 |
| **Total PA**  **(MET-hrs/wk)** ^a^ | 0.007 (0.018) | 0.040 | 0.692 | 0.003 (0.018) | 0.015 | 0.889 | -0.007  (0.020) | -0.034 | 0.739 | -0.010  (0.021) | -0.049 | 0.647 |

**^1)^** MET: metabolic equivalent task.

**^2)^** The R2 values for GAD-7 scores at 3-month and 6-month were 0.072 and 0.024, respectively. The R2 values for PHQ-9 scores at 3-month and 6-month were 0.050 and 0.011, respectively.

ILR, Intervention-Low Risk; BMI, body mass index; PA, physical activity; GAD-7, Generalized Anxiety Disorder 7-item scale; PHQ-9, Patient Health Questionnaire 9-item scale.
