# Supplementary material for: Changes in anxiety and depression levels and meat intake following recognition of low genetic risk for high body mass index, triglycerides, and lipoproteins: A randomized controlled trial
Source: PLoS One. 2023 Sep 8;18(9):e0291052. doi: 10.1371/journal.pone.0291052 (PMC10490956; doi:10.1371/journal.pone.0291052)
Supplement: S9 Table — 1) MET: metabolic equivalent task. 2) The R2 values for PHQ-9 scores at 3 months and 6 months were 0.197 and 0.055, respectively. ILR, Intervention-Low Risk; BMI, body mass index; PA, physical activity; PHQ-9, Patient Health Questionnaire 9-item scale. (DOCX) [file pone.0291052.s010.docx]

**S9 Table. Multiple linear analysis on the association between PHQ-9 and meat intake in the ILR group ^1), 2)^**

**^1)^** MET: metabolic equivalent task

**^2)^** The R^2^ values for PHQ-9 scores at 3 months and 6 months were 0.197 and 0.055, respectively.

ILR, Intervention-Low Risk; BMI, body mass index; PA, physical activity; PHQ-9, Patient Health Questionnaire 9-item scale

|  | **ILR group (n=32)** | | | | | | |
| --- | --- | --- | --- | --- | --- | --- | --- |
|  | **PHQ-9 score at 3-month** | | | **PHQ-9 score at 6-month** | | | |
|  | **B (SE)** | **Standardized *β*** | ***p*** | **B (SE)** | **Standardized *β*** | ***p*** |  |
| **Meat intake (g/d)** | -0.012 (01010) | -0.219 | 0.239 | -0.007 (0.010) | -0.156 | 0.492 |  |
| **Women vs. men** | 3.072 (2.169) | 0.314 | 0.169 | 1.050 (1.984) | 0.128 | 0.601 |  |
| **Age (years)** | 0.595 (0.387) | 0.275 | 0.136 | 0.320 (0.374) | 0.177 | 0.400 |  |
| **BMI (kg/m^2^)** | 0.167 (0.505) | 0.073 | 0.744 | 0.038 (0.460) | 0.020 | 0.935 |  |
| **Total PA (MET-hrs/wk)** ^a^ | 0.009 (0.035) | 0.048 | 0.794 | 0.000 (0.033) | 0.000 | 0.998 |  |
