# Supplementary material for: Changes in anxiety and depression levels and meat intake following recognition of low genetic risk for high body mass index, triglycerides, and lipoproteins: A randomized controlled trial
Source: PLoS One. 2023 Sep 8;18(9):e0291052. doi: 10.1371/journal.pone.0291052 (PMC10490956; doi:10.1371/journal.pone.0291052)
Supplement: S10 Table — 1) Change = metabolite level at follow-up–metabolite level at baseline. 2) One-way ANOVA tests and Kruskal-Wallis tests were used to determine the significant differences in changes in the metabolite levels among CON, INR, and IR groups. CON, control; ILR, Intervention-Low Risk; Intervention-High Risk. (DOCX) [file pone.0291052.s011.docx]

**S10 Table. Differences in relative levels of arginine and ornithine in the blood among CON, ILR, and IHR groups ^1), 2)^**

| **Total subjects (*n* = 100)** | | | | | | | |
| --- | --- | --- | --- | --- | --- | --- | --- |
| **Parameter** | **CON**  **(*n* = 35)** | | **ILR**  **(*n* = 32)** | | **IHR**  **(*n* = 33)** | | ***P* value ^2)^** |
|  | **Mean (SEM)** | **Change ^1)^** | **Mean (SEM)** | **Change ^1)^** | **Mean (SEM)** | **Change ^1)^** |  |
| **Arginine** | | | | | | | |
| Baseline | 6.9 (2.1) |  | 5.9 (2.3) |  | 6.2 (2.5) |  |  |
| 3-month follow-up | 5.3 (2.0) | -1.5 (2.3) | 11.4 (3.7) | 5.5 (4.3) | 5.9 (2.4) | -0.1 (2.2) | 0.426 |
| 6-month follow-up | 3.8 (1.7) | -3.0 (2.7) | 6.4 (2.3) | 0.5 (3.4) | 8.9 (2.7) | 2.6 (2.5) | 0.080 |
| **Ornithine** | | | | | | | |
| Baseline | 7.7 (5.6) |  | 5.4 (2.5) |  | 15.3 (6.9) |  |  |
| 3-month follow-up | 8.6 (3.0) | 0.8 (5.0) | 8.3 (3.3) | 2.9 (3.6) | 5.4 (3.0) | -9.9 (7.8) | 0.269 |
| 6-month follow-up | 4.6 (1.8) | -3.1 (5.0) | 1.5 (1.1) | -3.8 (2.6) | 7.2 (3.7) | -8.2 (7.5) | 0.395 |

**^1)^** Change = metabolite level at follow-up – metabolite level at baseline.

**^2)^** One-way ANOVA tests and Kruskal-Wallis tests were used to determine the significant differences in changes in the metabolite levels among CON, INR, and IR groups.

CON, control; ILR, Intervention-Low Risk; Intervention-High Risk
