# Supplementary material for: Changes in anxiety and depression levels and meat intake following recognition of low genetic risk for high body mass index, triglycerides, and lipoproteins: A randomized controlled trial
Source: PLoS One. 2023 Sep 8;18(9):e0291052. doi: 10.1371/journal.pone.0291052 (PMC10490956; doi:10.1371/journal.pone.0291052)
Supplement: S1 File — (DOCX) [file pone.0291052.s012.docx]

**STUDY PROTOCOL of DTC Genetic testing RCT**

**1. General Information**

**♦ Study Title:** Impact of DTC genetic information disclosure on psychological factors, dietary intake and health-related behavior

**♦ Name of the sponsor:** This research was supported by the grant from the Seoul National University Research Grant in 2018 (350-20180049)

**♦ Researcher Information**:

1) Principal investigator (PI):

Dr. Sung Nim Han, Department of Food and Nutrition, College of Human Ecology, Seoul National University, Seoul, Korea; [snhan@snu.ac.kr](mailto:snhan@snu.ac.kr)

2) Co-investigators:

Dr. Jung Han Kim, Department of Agricultural Biotechnology, Seoul National University, Seoul, Korea; [kjh2404@snu.ac.kr](mailto:kjh2404@snu.ac.kr)

Dr. Kyong-Mee Chung, Department of Psychology, Yonsei University, Seoul, Korea; [kmchung@yonsei.ac.kr](mailto:kmchung@yonsei.ac.kr)

3) Sub-investigators:

Ga Young Lee, Department of Food and Nutrition, College of Human Ecology, Seoul National University, Seoul, Korea; [lgykiki90@snu.ac.kr](mailto:lgykiki90@snu.ac.kr)

Jung Hak Kim, Department of Agricultural Biotechnology, Seoul National University, Seoul, Korea; [crane245@snu.ac.kr](mailto:crane245@snu.ac.kr)

**♦ Trial registration:** Clinical Research Information Service (CRIS), KCT0004650 [https://cris.nih.go.kr/cris/search/detailSearch.do /14091](https://cris.nih.go.kr/cris/search/detailSearch.do%20/14091)

**2. Purpose and background of the study**

**2.1 Purpose of the study**

This study is a preliminary study investigating the impact of recognizing direct-to-consumer (DTC) genetic testing (GT) results on changes in psychological factors, dietary intake and physical activity. This study aims to lay the foundation for precision nutrition, which is a nutrition management that considers genetic factors for prevention and management of diseases, by comprehending the changes in metabolites and health-related behaviors following recognition of genetic information. DTC-GT provides information on general wellness area such as body mass index (BMI), triglycerides and cholesterol concentration, blood glucose levels, blood pressure, and pigmentation, hair loss, thickness, skin aging, elasticity, vitamin C concentration, and caffeine metabolism. If assigned to a control group, subjects will be informed about their genetic test results at six months of follow-up, after all tests are completed.

This study 1) analyzes the effects of genetic factors on taste sensitivity and dietary intake, 2) examines the impacts of genetic information disclosure on changes in psychological factors, dietary intake, and physical activity, and 3) identifies changes in health-related behavior following genetic information recognition and verify changes in blood metabolites.

**2.2 Background of the study**

This study is presumed to be the first study attempted in Korea. In Korea, several studies that investigated the relationships between diseases and the causes of those diseases have been conducted, but study that examined the effects of recognizing GT results on various aspects of health-related behavior is limited. The genetic test items that can be tested and results being directly notified to the subjects by GT institutions are listed on the Ministry of Health and Welfare notice (No. 2016-97). These items include the BMI, triglycerides, cholesterol, blood glucose, blood pressure, vitamin C, and caffeine metabolism, hair loss, skin aging, BMI, cholesterol, and blood pressure. The Korean Ministry of Health and Welfare held a public hearing on April 30^th^ of 2018 in order to collect opinions on how to improve the DTC-GT regulatory systems, which suggests the interest on DTC and possibility of changes in regulation. However, impact of recognizing the DTC results on psychological aspects and health-related behavior of the consumers has been overlooked and more research is needed.

**2.2.1 The necessity for precision nutrition research**

Precision medicine, which is a personalized medicine that considers personal diversity such as gene, environment, and lifestyle, has been emerged as a treatment strategy for diseases. Precision nutrition is essential for precision medicine because dietary factors play an important role in prevention and management of various diseases. Precision nutrition is particularly important in terms of preventing chronic diseases and can be applied to the treatment of diseases that require nutritional management. If diet and health-related activities are modified in a positive direction before the onset of the disease, it is expected to have a significant preventive effect. Therefore, this study may help lay the foundation for a successful application of precision nutrition that can contribute to a healthy lifestyle, given the significance of precision nutrition in terms of disease prevention.

**2.2.2 The necessity for convergence research**

The intervention plan for the effective implementation of precision nutrition should be based on the risk of disease based on genetic factors, and it should be considered that individuals respond differently to dietary intake depending on the genotype. In addition, for precision nutrition to be effectively implemented, changes in the subjects’ dietary habits and health-related behaviors should be achieved. Precision Nutrition must be approached from various aspects and consideration for the psychological factors which is important for understanding behavioral changes.

**2.2.3 Effects of recognition of genetic information on health-related behavior**

The recognition of genetic information did not significantly motivate risk-reducing behavior according to a meta-analysis of 18 studies (6 on quitting smoking, 7 on a diet, and 6 on physical activity) [Hollands GJ 2016]. However, the psychological and behavioral patterns of Koreans seem to be different from those of Westerners. Considering this, there is a need for high-quality research on Koreans to determine whether understanding genetic information motivates risk-reducing behavior.

**2.2.4 Effects of genetic factors on taste sensitivity or perception**

Previous research has suggested that genetic factors affect taste sensitivity or perception. According to a previous study, the *TRPV1* and *SCNN1B* genes have been reported as genes affecting perception of saltiness, and people with different genotypes have been shown to perceive saltiness differently [Dias AG 2013]. The difference in sensitivity to sweetness and sugar is also found to vary depending on BMI in the case of *TAS1R2* [Dias AG 2015]. Since taste perception and sensitivity is a decisive factor in food choice, it is necessary to understand how genetic factors affect taste perception in order to change dietary behaviors.

**2.2.5 A study on genetic factors and dietary consumption**

Genetic factors can affect the intake of certain nutrients. It has been reported that the polymorphism of *AMY1* affects dietary intake patterns [Sorkin R 2017]. In Westerners, the A allele carriers of rs11185098 in the *AMY1* gene showed high intakes of total carbohydrates, while in Asians, subjects with A genotype of the rs1999478 showed high intake of calories and sugar. Personalized nutritional management can be reinforced by considering not only the relationship between genetic factors and disease risk but also the relationship between genetic factors and nutrient intake.

**2.2.6 Psychological factors and dietary intake**

Since psychological factors affect food choice and fidelity to the intervention, a thorough understanding of psychological factors is required for effective nutritional intervention. It has been reported that the preference for high energy, sugar-rich, and fatty foods increases in long-term stress situations (Torres SJ 2007). In addition, a negative relationship has been reported between the Mediterranean Diet Score and depression and anxiety levels (Hodge A 2013). Furthermore, it was found that individual personalities affect dietary intake; those with openness consume more vegetables and fruits, and those with conscientiousness have a low risk of obesity due to more desirable behaviors in health-related behaviors [Lunn TE 2014]. However, since most of the previous studies were conducted on Westerners, there is a limit to apply the results to Koreans. Koreans differ from Westerners and other Asians in their food preferences and tastes. Therefore, more studies are needed to verify the relationship between psychological factors and the dietary intake of Koreans.

**2.2.7 Essential factors for the development and application of precision nutrition and the necessity of evidence for the establishment of precision nutrition for Koreans**

Interaction between genetic factors and nutrients play an important role in personal health. General nutritional management guidelines were used in the past, however, access to genetic information has become easier, and intervention considering individual diversity is possible these days. Genetic factors influence numerous diet-related diseases and also affect individual’s responses to nutritional interventions. Therefore, for the development of precision nutrition, it is necessary to understand the interrelationship between diet, genes, and health and accumulate sufficient scientific evidence. Additionally, although there are genetic factors affecting clinical or health indicators that are common between Westerners and Asians, the frequency of genotypes varies by race. Since the purpose of precision nutrition is a nutrition intervention considering individual diversity, acquiring the results on Koreans is essential for implementation of precision nutrition for Koreans.

Therefore, in this study, it is anticipated to induce study results that can be practically applied to precision nutrition suitable for Koreans by investigating behavioral changes following recognition of genetic information and analyzing factors that induce behavioral changes.

**3. Objectives**

This study will investigate the impact of DTC-GT results recognition on psychological factors and changes in dietary intake and physical activity. We intended to contribute to lay the foundation for the successful implementation of precision nutrition by investigating changes in health-related behaviors and quality of life following genetic information recognition.

**4. Criteria for selection and exclusion of study subjects**

**4.1 Subject inclusion criteria**

- Adults aged 25-35 years old

- BMI of 18.5 to 25 kg/m^2^

- Healthy people without any diseases

- Voluntarily agreed to participate in the study

**4.2 Subject exclusion criteria**

- Those who have been diagnosed with or have been treated for cancer

- Those who have diabetes, heart disease, kidney disease, lung disease, high blood pressure, and excessive allergic reactions

- Those who are pregnant or plan to be pregnant within a year

- Graduate students who belong to the laboratory of the research PI or students who take the course lectured by the research PI

**4.3 Number of target study subjects and basis for calculation**

The final number of the study subjects were aimed to be a total of 100 people, of which 65 belonged to the intervention group and 35 belonged to the control group. There was a meta-analysis of 18 previous studies that have investigated the relationship between predicting disease risk based on genetic information and health-related behavioral changes, of which 7 studies were related to dietary intake and 6 studies were related to physical activity (Hollands GJ 2016). These studies included at least 107 to 601 study subjects. Furthermore, a study that have examined the relationship between genetic differences and taste sensitivity included a total of 95 subjects, and it was shown that the threshold for saltiness was 173 % (A>T) higher for the carriers of A allele of the SNP rs239345 compared to those with T allele and 160 % (T>C) higher for subjects with T allele of the rs3785368 than those who carry C allele (Dias AG et al, 2012).

This study aims to lay the foundation for the effective implementation of precision nutrition to Koreans, and has the characteristics of a preliminary study. Although it is not a large-scale population study, a total number of subjects were set to 100 in consideration of a dropout rate of around 10% as in previous related studies (Nielsen D & El-Sohemy, 2014 & Dias AG et al, 2012).

**5. Comparison of group settings**

The ratio of the subjects in the intervention group and the control group was set to 2:1 on the premise that response variability after genetic information recognition will be high in the intervention group. A random allocation method was used to divide the intervention and the control groups.

- Intervention group: 65 subjects who are recognized with their genetic information

- Control group: 35 subjects who are unaware of their genetic information

**6. Research design**

**6.1 Subject recruitment**

The recruitment of study subjects will be conducted offline (public notice on the Seoul National University bulletin boards) and online (the Seoul National University portal site) using a recruitment notice which was approved by the Institutional Review Board (IRB). The subjects who met the recruitment conditions of the study are healthy adults aged 25 to 35 who voluntarily expressed interest in participation, with a BMI of 18.5 to less 25 kg/m^2^.

**6.2 Proceedings of the research consent process**

The researchers will conduct an orientation before the start of the study explaining the details of the study and the consent form to the participants. The subjects will sign a consent form approved by the IRB if their willingness to participate does not change. Graduate students affiliated with the research PI or who attend the course lectured by the research PI will excluded from the study.

**6.3 Practical research process**

**6.3.1 Blood collection and clinical examination**

After 12 hours of fasting, 11 mL of blood will be taken from subjects for genetic information analysis, metabolites measurements, and evaluation of clinical indicators. Blood will be drawn at the baseline, 3 months, and 6 months follow-ups, and a clinical examination will be conducted at the Seoul National University Health Service Center. Clinical indicators such as complete blood count, anemia index, fasting blood glucose, total serum cholesterol, triglycerides, low-density lipoprotein and high-density lipoprotein-cholesterol, and liver function variables will be examined using 5 mL of the whole blood. 3 mL of blood is used for the genetic analysis, and the remaining 3 mL of blood is used for metabolite analysis and further measurement.

**6.3.2 Anthropometric measurement**

The anthropometric measurement will be conducted at the Department of Food and Nutrition at Seoul National University (Building 222, Room 604). Body weight, muscle mass, and fat mass will be measured by using Inbody 720 (Biospace Co. Ltd.). Height and waist circumference will be measured to confirm the BMI and abdominal obesity. Anthropometric indicators are measured at baseline and 1-, 2-, 3-, and 6-month follow-ups.

**6.3.3 DTC genetic testing**

The genetic analysis will be conducted by Teragen ETex (TheragenEtex Inc., Suwon, Korea) using blood drawn from the baseline. The Theragen Precision Medicine Research Array (PMRA) chip that will be used in this study is based on approximately 750,000 single nucleotide polymorphisms of the Axiom-based Asian PMRA chip developed by Thermo Fisher and is known to be suitable for use in research for various research purposes. According to guidelines established by the Ministry of Health and Welfare (No. 2016-97), GT provides subjects with genetic information on a total of 12 items (BMI, triglycerides concentration, cholesterol, blood glucose, blood pressure, pigmentation, hair loss, hair thickness, skin aging, skin elasticity, vitamin C concentration, and caffeine metabolism).

**6.3.4 Metabolite analysis**

Blood metabolites will be analyzed by GC-MS/MS method using 50 µL of serum from the pesticide chemistry and toxicology laboratory of the Department of Agricultural Biotechnology at Seoul National University. 304 markers, including monosaccharides, amino acids, fatty acids, nucleic acids, cholesterol, sugar alcohol, metabolic intermediates (TCA cycle, glycolysis, urea cycle, etc.), and vitamins (biotin and vitamin C) will be measured after the derivatization by methoxidation/trimethylsilylation. Seven short-chain acid species will be analyzed after derivatization with 4,6-dimethoxy-1,3,5-triazin-2-yl-4-methylmorpolinium chloride and n-octylamine, using acetic acid as internal standard. The metabolites will be measured at the baseline and 3- and 6-month follow-ups.

**6.3.5 Measurement of taste sensitivity**

The subjects will be exposed to various concentrations of sugar and sodium chloride to test their sensitivity to sweetness and saltiness. They will then fill out a 3-AFC questionnaire to indicate when they first felt the signal. Using six concentrations of sugar (sucrose: 0.40, 10.55, 20.70, 30.85, 41.00, and 51.00 g/L) and six concentrations of salt (sodium chloride: 0.006, 0.011, 0.445, 0.380, 1.315, and 1.750 g/L) diluted with distilled water, the sensitivity to sweetness and saltiness will be measured by providing them in the order in which the concentration increases. Study subjects will rinse their mouths with fresh water between each concentration and check the point at which the difference in taste is felt. The measurement of the sensitivity to sweetness and saltness will be performed at the baseline

**6.3.6 Dietary and nutrient intake**

The Food Frequency Questionnaire (FFQ, National Health, and Nutrition Survey) will be used to measure long-term dietary intake and dietary records will be used to measure short-term dietary intake. A three-day dietary record includes two weekdays and one weekend. A clinical dietitian will examine the subjects' FFQ and dietary records on the day of the visit to confirm accuracy. The Computer-Aided Nutritional Analysis Program 5.0 will be used to analyze the FFQ at the time of baseline, 3- and 6-months follow-ups, and for dietary records at the baseline, 1-, 2-, 3-, and 6-month follow-ups.

**6.3.7 Assessment of psychological factors**

Depression/Anxiety (PHQ-9, GAD-7), health-related quality of life (SF-36), self-esteem (Rosenberg's Self-esteem Scale), and impulsivity (BIS-11) will be administered online through the survey monkey website (<https://ko.surveymonkey.com>). At the baseline, 3- and 6-month follow-ups, a delay discounting task that assesses the capacity to postpone satisfaction will be used to measure impulsivity further (Chol BY. 2011).

**6.3.8 Measurement of changes in physical activity**

The physical activities including distance, number of steps, calorie consumption, and activity time and change in sleep duration will be measured for 10 days before visits at baseline, 1-, 2-, 3-, and 6- months using a wearable device (Fitbit charge 2). Additionally, online surveys will be conducted using the questions from the International Physical Activity Questionnaire-short form, a physical activity survey that divides questions into categories based on the level of exercise.

**6.4 Information related with questionnaires**

**6.4.1 Basic personal information of study subjects**

In this study, researchers will allocate a random subject ID number at the beginning of the study to avoid the exposure of study subjects’ personal information. Therefore, all questionnaires used in the study do not require any personal information other than the subject ID number. However, the contact person needed to know the subject’s name and phone number. The subject ID number will be managed using a barcode, and a person who does not directly meet the subjects will match the subject ID numbers with the subjects’ personal information.

**6.4.2 Type and content of the questionnaires**

• Questionnaire related to psychological factors

- PHQ-9: A questionnaire that evaluates a major depressive disorder consisting of nine items

- GAD-7: A questionnaire that evaluates a generalized anxiety disorder consisting of seven items

- SF-36: A questionnaire that evaluates health-related quality of life consists of nine questions on physical and mental conditions and their effect on daily activities

- Rosenberg self-esteem scale: A questionnaire consisting of ten questions that evaluates how much value people give themselves

- BIS-11: A questionnaire consisting of 16 questions as an evaluation for impulsivity

• Questionnaire related to taste sensitivity

- 3-AFC: A test to determine the sensitivity of the taste by finding one different sample with the highest intensity for a particular characteristic among the three samples.

• Questionnaire related to physical activities

- IPAQ-SF: A questionnaire that evaluated activity time and frequency of physical activity consisting of 7 items.

**7. Disposal of laboratory equipment using blood**

The IRB's legal management format for the human-derived materials will be used for this study. Experiment equipment used with blood will be separately discarded in accordance with the biohazard waste treatment regulations. Subject ID will be removed to prevent sample identification. At the time of disposal, the blood sample used for the clinical evaluation will be recorded in the disposal records.

**8. Statistical analysis**

Continuous variables will be tested for normality and those that do not comply with normality assumptions will be log-transformed prior to analysis to improve the normality. Repeated measures will be used in statistics to analyze the changes in each variable from baseline to follow-up time-points. An independent *t*-test will be used to compare the differences in variables between the intervention and control groups and a paired *t*-test will be used to compare before and after the intervention within each group.

**9. Safety considerations for research subjects**

The test results from this study will not be used in any other way except for the investigation of the "Impact of DTC genetic information disclosure on psychological factors, dietary intake, and health-related behavior." An expert at the Seoul National University Health Service Center will inform the subjects about potential side effects during the blood collection, such as lightheadedness, bruises, shocks, etc. If such side effects occur, the research manager and PI will be in charge of the treatment process and take the necessary measures such as first aid (hemostasis, cold compress, etc.) immediately.

If study subjects no longer want to participate the study, they can stop at any point during the study period. There will be no negative impact on the subjects even if they decide not to participate continuously. Non-identification information, such as gender and age can be used for analysis even after subjects stop participating in the study. Still, all personal information, including names and contact information, will be immediately discarded and deleted.

**10. Research schedule**

Total Study Period: 2018.8.1-2019.7.31

1) 2018.8 ~ 2018.11

: Apply for IRB approval and establish and review the research methods

2) 2018.12 ~ 2019.5

- DTC genetic testing and measurement of taste sensitivity: Baseline

- Measurement of anthropometric data, physical activity, and dietary intake: Baseline, 1, 2, 3, 6 months follow-ups

- Psychological evaluation, clinical examination, metabolic measurement: Baseline, 3, 6 months follow-ups

3) 2019.6 ~ 2019.7

: Organize research results and write report and papers

**11. Ethical considerations in the research**

The personal information of the subjects collected at the beginning of the study will be kept in a location accessible only to the research PI so that the rights and interests of the subjects are respected to the greatest extent as possible. All the documents containing personal information obtained for payment will be destroyed using a paper shredder at the end of the study. Electronic files will be irreversibly and permanently deleted. A code number and barcode system that is randomly assigned to each subject will be utilized by the research manager to carry out the study. Barcode and code numbers will also be used in all online and documentary questionnaires provided to study subjects to minimize the exposure of subjects' personal information.

All the study procedures will be carried out solely within the parameters established by the applicable regulations and will not be revealed for purposes other than study analysis. The IRB-approved consent form will be used to obtain the study subject's consent to participate in the experiment. Study subjects will receive adequate explanations of the research contents and the consent form. If their intentions to participate in the study have not changed, they will proceed to sign the consent form. The research consent form will be destroyed three years after the study is completed, but the research materials can be kept as long as possible based on the research ethics guidelines of the Seoul National University.

The blood sample in this study will be used for clinical measurement, genetic information analysis, and evaluation of metabolites, and the results will only be notified to subjects if they agree to be provided. Additionally, following Article 39 (1) of the Bioethics and Safety Act, human derivatives will be disposed of immediately after the storage period ends by applying the statement in the legal form of the human derivatives research consent. Blood samples will be managed and tracked in the storage ledger and kept in a deep freezer in room 623 of building 222. The PI and the research manager of the study will be responsible for the samples' use, storage, and treatment.

**11. References**

Hollands GJ, French DP, Griffin SJ, Prevost AT, Sutton S, King S et al. The impact of communicating genetic risks of disease on risk-reducing health behaviour: systematic review with meta-analysis. BMJ. 2016;352:i1102

Dias AG, Rousseau D, Duizer L, Cockburn M, Chiu W, Nielsen D et al. Genetic variation in putative salt taste receptors and salt taste perception in humans. Chem Senses. 2013;38(2):137-45

Dias AG, Eny KM, Cockburn M, Chiu W, Nielsen DE, Duizer L et al. Variation in the TAS1R2 Gene, Sweet Taste Perception and Intake of Sugars. J Nutrigenet Nutrigenomics. 2015;8(2):81-90

Sorkin R, Wolever T, El-Sohemy A. Genetic variation in the AMY1 gene is associated with dietary carbohydrate and starch intake in a young adult population. FASEB J 2017;31:S299.5

Torres SJ, Nowson CA. Relationship between stress, eating behavior, and obesity. Nutrition. 2007 (11-12):887-94

Hodge A, Almeida OP, English DR, Giles GG, Flicker L. Patterns of dietary intake and psychological distress in older Australians: benefits not just from a Mediterranean diet. Int Psychogeriatr. 2013;25(3):456-66

Lunn TE, Nowson CA, Worsley A, Torres SJ. Does personality affect dietary intake? Nutrition. 2014;30(4):403-9

Chol BY and Chung KM. Utility of Delay Discounting Task as a Measure of Impulsivity. Kor J Psychol. 30(4), 2011.11

Stewart-Knox BJ, Simpson EE, Parr H, Rae G, Polito A, Intorre F et al. Zinc status and taste acuity in older Europeans: the ZENITH study. Eur J Clin Nutr. 2005;59 Suppl 2:S31-6

Nielsen DE and EL-Sohemy A. Disclosure of Genetic Information and Change in Dietary Intake: A Randomized Controlled Trial. PLoS One. 2014 Nov 14;9(11)
