# Supplementary material for: Changes in anxiety and depression levels and meat intake following recognition of low genetic risk for high body mass index, triglycerides, and lipoproteins: A randomized controlled trial
Source: PLoS One. 2023 Sep 8;18(9):e0291052. doi: 10.1371/journal.pone.0291052 (PMC10490956; doi:10.1371/journal.pone.0291052)
Supplement: S2 File — (DOCX) [file pone.0291052.s013.docx]

**연구계획 심의의뢰서(접수번호: SNU 18-10-004)**

**♦ 과제명: DTC 유전자검사 결과의 인지가 심리적 영역과 식생활 및 건강관련 행동에 미치는 영향**

**♦ 연구비 지원 기관: 서울대학교(350-20180049)**

**♦ 연구자 정보**

**1) 연구책임자:**

**한성림, 교수, 서울대학교 생활과학대학 식품영양학과;** [**snhan@snu.ac.kr**](mailto:snhan@snu.ac.kr)

**2) 공동연구자:**

**김정한, 교수, 서울대학교 농업생명과학대학 농생명공학부;** [**kjh2404@snu.ac.kr**](mailto:kjh2404@snu.ac.kr)

**정경미, 교수, 서울대학교 연세대학교 심리학과;** [**kmchung@yonsei.ac.kr**](mailto:kmchung@yonsei.ac.kr)

**3) 연구담당자:**

**이가영, 서울대학교 생활과학대학 식품영양학과;** [**lgykiki90@snu.ac.kr**](mailto:lgykiki90@snu.ac.kr)

**김정한, 서울대학교 농업생명과학대학 농생명공학부;** [**crane245@snu.ac.kr**](mailto:crane245@snu.ac.kr)

**♦ 임상연구 등록: Clinical Research Information Service (CRIS), KCT0004650** [**https://cris.nih.go.kr/cris/search/detailSearch.do /14091**](https://cris.nih.go.kr/cris/search/detailSearch.do%20/14091)

**2. 연구 목적 및 배경**

**2.1 연구 목적**

본 연구는 소비자 의뢰 유전자 검사 (DTC) 결과를 통한 유전정보의 인지가 심리적 요인 및 식생활과 신체활동의 변화에 미치는 영향을 알아보기 위한 preliminary study로써, 유전정보의 인지로 인한 건강 관련 행동과 삶의 질 변화를 파악하고 체내 대사체의 변화를 확인하여 질환의 예방 및 관리의 측면에서 유전적 요인을 고려한 영양관리를 의미하는 정밀영양 (Precision Nutrition)의 적용을 효과적으로 할 수 있는 기초를 마련하기 위한 연구임. 유전자 검사는 연구담당자가 유전자검사 전문기관에 의뢰하는 형식으로 진행되며, 체질량지수, 중성지방농도, 콜레스테롤, 혈당, 혈압, 색소 침착, 탈모, 모발 굵기, 피부 노화, 피부 탄력, 비타민 C 농도, 카페인 대사에 해당하는 12가지 항목에 대한 유정 정보를 제공함. 대조군 (유전정보 미인지군)으로 배정된 경우 마지막 방문인 6개월 시점에서 모든 검사가 종료된 후 해당 정보를 알려줌.

따라서, 본 연구는 1) 유전적 요인이 맛에 대한 민감도 및 식이 섭취 (영양소 섭취)에 미치는 영향을 분석하고, 2) 소비자 의뢰 유전자 검사 (DTC, Direct to consumer) 전정보의 인지가 심리적 요인, 식생활, 신체활동의 변화에 미치는 영향을 알아봄으로써 3) 유전정보의 인지로 인한 삶의 질 및 건강 관련 행동의 변화를 파악하고 임상지표 및 체내 대사체의 변화를 확인하고자 함.

**2.2 연구 배경**

본 연구는 국내에서는 처음으로 시도되는 연구로 파악되고 있음. 유 과의 관계에 대한 한국인 대상 연구는 비교적 활발하게 이루어지고 인지가 미치는 영향을 다각적인 측면에서 알아본 연구는 없음. 현재 유전적 요인과 질병 있으나, 유전정보의 일부 만성질환 관련 유전정보의 검사 및 검사결과 확인이 일반인에게도 가능하게 되었음. 유전자 검사기관에서 직접 실시하여 검사 대상자에게 알려줄 수 있는 항목은 보건복지부고시(제2016-97호)로 정해져 있으며, 체질량지수, 중성지방농도, 콜레스테롤, 혈당, 혈압, 비타민 C, 카페인대사, 탈모, 모발 굵기, 피부노화 관련 유전자임, 이중 체질량지수, 중성지방농도, 콜레스테롤, 혈당, 혈압은 그 조절에 있어서 식이 요인이 중요한 역할을 보건복지부에서는 2018년 4월 30일에 'DTC 유전자 검사 제도개선'할 수 있는 지표임. 공청회를 열어 '소비자 의뢰 유전자검사 (DTC)'의 활성화를 위한 규제 개선 방안에 대해 각계의 의견을 수렴하는 자리를 마련하여 DTC 유전자검사제도에 대한 관심과 제도의 변화 가능성을 시사하였으나, 유전자 검사의 결과가 소비자에게 주는 심리적 영향 및 건강관련 행동에 미치는 영향은 간과되고 있어 이 부분에 대한 연구가 시급함.

**2.2.1 정밀영양 연구의 필요성**

정밀의학 또는 정밀 의료 (Precision medicine)는 유전적 요인과 질환 위험도의 관련성을 밝혀 개인적인 다양성을 고려한 예방과 치료 전략으로 부상하고 있음. 많은 질환의 예방 관리에서 영양적 요인은 중요한 부분을 차지하고 있기에 정밀 의료를 위해서는 정밀영양 (Precision nutrition)을 간과할 수 없음. 특히, 만성질환의 예방적인 측면에서는 정밀영양의 역할이 크며, 영양소 대사에 영향을 미치는 유전적 요인이나 유전적 요인이 변화를 일으키는 임상변화 중 영양관리가 필수적인 지표의 경우 정밀영양의 적용이 중요한 역할을 함. 유전적 요인을 파악하여 질환으로 발전되기 전에 식습관, 영양소 섭취, 건강관련 활동을 바람직한 방향으로 수정할 경우 예방효과가 클 것임. 따라서 질환의 예방적 측면에서 정밀영양의 중요성을 고려하였을 때 본 연구를 통해 건강한 수명유지에 기여할 수 있는 효과적인 정밀영양 적용 방안의 토대를 구축할 수 있을 것으로 기대됨.

**2.2.2 융복합 연구의 필요성**

정밀영양의 실행이 효과적으로 이루어지기 위한 중재 계획은 유전적 요인으로 인한 질환의 위험도에 근거하고, 유전자형 별로 식이에 대해 다르게 반응하며, 대상자가 수용하고 실행하는 행동변화까지 이루어져야 함. 따라서, 정밀영양의 실행이 효과적으로 이루어지기 위해서는 대상자의 식생활 변화 및 건강 관련 행동의 변화가 일어나야 하며, 행동변화에 대한 해를 위해서 심리적 요인까지 고려한 다각적인 측면에서 정밀영양 및 맞춤형 영양이 접근되어야 함.

**2.2.3 유전정보의 인지가 건강관련 행동에 미치는 영향**

18개의 연구(금연 6개, 식이 7개, 신체활동 6개)를 메타분석한 결과에 의하면 유전정보의 인지가 위험도를 감소시키는 행동을 유도하는 동기부여 효과는 크지 않은 것으로 나타났으나 [Hollands GJ 2016], 대체적으로 연구의 수준이 높지 않다는 평가가 있음. 또한 한국인의 심리적 및 행동 양상은 서양인과 다르게 나타남. 이를 고려했을 때 유전정보의 인지가 위험도를 저하시키는 행동에 대한 동기부여가 되는지에 대한 연구는 아직 미성숙한 단계이므로, 한국인을 대상으로 한 수준 높은 연구의 필요성이 있음.

**2.2.4 유전적 요인이 맛에 대한 민감도 또는 인식에 미치는 영향**

선행연구에 의하면 유전적요인이 맛에 대한 민감도나 인식에 영향을 미친다는 것이 보 고됨. 짠맛에 대한 인식에 영향을 주는 유전자로는 *TRPV1*과 *SCNN1B* 유전자가 보고되었는데, 각 유전자형에 따라 짠맛에 대한 강도를 느끼는 정도가 달랐음이 보고됨 [Dias AG 2013]. 또한, 단맛에 영향을 주는 유전자인 *TAS1R2*의 경우 유전자형에 따른 단맛에 대한 민감도 및 설탕 섭취량의 차이는 체질량지수에 따라 다르게 나타남이 확인됨. [Dias AG 2015]. 맛에 대한 인식은 식품 선택에 있어서 중요한 요인이므로 식생활 변화를 유도하기 위해 유전적 요인이 맛에 대한 민감도나 인식에 미치는 영향에 대한 깊은 이해가 필 요함.

**2.2.5 유전적 요인과 식이 섭취에 대한 연구**

유전적 요인이 특정 영양소의 섭취량에 영향을 줄 수 있다는 연구결과가 보고되고 있음. *AMY1* 유전자의 다형성은 식사섭취 패턴에 영향을 미치는 것으로 나타났는데 [Sorkin R 2017], 서양인에서는 *AMY1* 유전자의 rs11185098의 A 유전자형이 총 탄수화물 섭취량이 높은 것으로 나타났으며, 동양인에서는 rs1999478의 A 유전자형이 에너지와 설탕의 섭취가 높은 것으로 나타났음. 이러한 유전적 요인과 식이 섭취에 대한 연구를 통해 유전적 요인과 질환의 위험도뿐만 아니라 유전적 요인과 영양소 섭취와의 관계까지 고 려한 접근은 맞춤형 영양관리를 강화할 수 있을 것임.

**2.2.6 심리적 요인과 식이 섭취**

심리적 요인은 식품의 선택 및 식행동에 영향을 미칠 뿐만 아니라 중재에 대한 충실도에도 영향을 줄 수 있으므로, 효과적인 영양중재를 위해서는 심리적인 요인에 대한 심도 있는 이해가 필요함. 선행연구에 따르면, 장기간의 스트레스는 에너지가 많고 설탕과 지방이 많은 식품에 대한 선호도와 관련이 있다고 보고되었으며 [Torres SJ 2007], 식사 섭취를 조사한 후 12년 후에 우울과 불안 정도를 측정한 연구결과에 의하면 지중해식 식사 점수 (Mediterranean Diet Score)와 정신적 고통 간에는 음의 상관관계를 보여줌 [Hodge A 2013]. 또한, 개인의 성향도 식이 섭취 및 식이 중재에 영향을 미치는 것으로 보이는데, 개방성 (openness) 성향을 가진 사람이 채소와 과일의 섭취가 많고, 신중형 (conscientiousness)인 사람은 건강 관련 행동에 있어서 더 바람직한 행동을 하며 비만 위험도가 낮은 것으로 나타남 [Lunn TE 2014]. 그러나, 이러한 연구들은 모두 서양인 대상 연구이므로 한국인에게 적용하는데 있어서 한계가 있음. 식사패턴이나 맛에 대한 선호도에 대하여 한국인은 서양인이나 다른 동양권 사람들과 차이를 보임. 따라서 한국인을 대상으로 심리적 요인과 식이 섭취의 관련성을 확인하는 연구가 필요함.

**2.2.7 정밀영양의 발전 및 적용을 위한 필수요인 및 한국인을 대상으로 한 정밀영양의 확립을 위한 근거자료의 필요성**

개인의 건강에는 유전적 요인과 영양적 요인 간의 상호작용이 중요함. 일반적인 영양 중재 관리지침을 사용했던 과거와는 달리 유전정보에 대한 접근이 용이해짐에 따라 개 인적 다양성을 고려한 중재가 가능해지게 되었음. 유전적 요인은 식생활과 관련된 많은 질환들에 영향을 주는데, 동시에 영양중재에 대한 반응 또한 유전적 요인의 영향을 받 기도함. 따라서 정밀영양의 발전을 위해서는 충분한 과학적 근거의 축적 및 이해가 필 요함. 따라서, 식이-유전자-건강 간의 상호관계에 대한 과학적 지식이나 근거 확립이 필 요함. 또한, 일부 임상 또는 건강지표에 영향을 미치는 유전적 요인은 서양인과 동양인 에게 공통으로 나타나기도 하나, 많은 유전형의 빈도에 있어 인종에 따라 차이가 있음. 정밀영양의 기본 취지는 개인적 다양성을 고려한 영양 중재이므로, 한국인을 대상으로 한 연구 결과의 확보는 한국인을 위한 정밀영양을 위해 필수적임.

결과적으로 본 연구를 통해 유전정보의 인지로 유도되는 행동변화를 알아보고, 행동 변 화를 유도하는 요인을 분석함으로써 한국인에게 맞는 정밀영양에 대한 실제적인 효용 성을 제공할 수 있는 연구결과가 도출될 것으로 기대함.

**3. 연구목표**

소비자 의뢰 유전자 검사 (DTC) 결과를 통한 유전정보의 인지가 심리적 요인 및 식생 활과 신체활동의 변화에 미치는 영향을 알아봄으로써 유전정보의 인지로 인한 건강 관련 행동과 삶의 질 변화를 파악하고 체내 대사체의 변화를 확인하여 정밀영양 (Precision Nutrition)의 적용을 효과적으로 할 수 있는 기초를 마련하고자 함.

**4. 연구참여자의 선정기준과 제외기준**

**4.1 연구참여자의 선정기준**

- 만 25-35세의 성인

- BMI가 18.5 이상 25 kg/m^2^ 미만인 사람

- 질병이 없는 건강한 사람

- 자의로 연구 참여를 결정하고 연구 참여에 동의한 사람

**4.2 연구참여자의 제외기준**

- 암을 진단받았거나 치료를 받은 경험이 있는 사람

- 현재 당뇨병, 심장질환, 신장질환, 폐질환, 고혈압 등이 있거나, 과도한 알레르기 반응이 있는 사람

- 임신 중이거나 1년 이내에 임신을 계획하고 있는 사람

- 연구책임자의 실험실 소속 대학원 학생이나 강의 수강생

**4.3 목표 연구참여자의 수 및 산출 근거**

최종 연구 대상자는 총 100명으로서 실험군 65명, 대조군 35명을 목표로 함. 유전자를 토대로 질병 위험도 예측과 위험도 감소를 위한 건강 관련 행동 및 참여 동기의 관련성에 대한 18개의 연구를 메타 분석한 선행연구에서는 식이에 관련하여 7개 연구를 통합하여 총 1784명의 참여자를 대상으로 하였고, 신체활동에 관련해서는 총 6개의 연 구를 1704 명의 참여자를 대상으로 분석하였음 [Hollands GJ 2016]. 식이 및 신체활동 관련 각 연구를 살펴보았을 때 최소 107명, 최대 601명의 연구 참여자를 대상으로 하였음. 참고한 선행연구 중 중재연구는 아니었으나 맛의 민감도에 대한 연구(Dias AG et al, 2012)에서는 총 연구 참여자가 95명이었음에도 짠맛 관련 유전자인 rs239345와rs3785368의 SNP 중 각각 A와 T allele에 대하여 평균 173% (A>T), 160% (T>C) 수준으로 짠맛에 대한 역치가 높아 유의적인 차이를 확인하였음. 본 연구는 한국인을 대상으로 정밀영양의 적용을 효과적으로 할 수 있는 기초를 마련하고자 하는 연구로써 Preliminary test의 성격을 가짐. 따라서 유전자 검사의 인지가 사람 들의 행동 변화에 미치는 영향을 확인하기 위해 한국인을 대상으로 한 연구 결과의 확보 및 축적을 위한 하나의 연구로 계획됨. 본 연구는 대규모의 참여자를 대상으로 한 연구는 아니지만 피험자의 수는 선행된 관련 연구(Nielsen D & El-Sohemy A, Disclosure of Genetic Information and Changes in Dietary Intake: A Randomized Controlled Trial, 2014)와 유사하게 약 10%의 탈락율을 고려하여 총 모집 연구 참여자를 100명으로 설정하였음.

**5. 비교군 설정**

- 실험군: 유전정보 인지군 65명

대조군 (Control): 유전정보 미인지군, 35명으로서 실험군과 매치 (match)하여 선정함.

- 실험군과 대조군의 선정:

1) 유전정보 인지 시 반응의 다양성 (Variability)이 미인지군의 변화에 비해 클 것이라는 가정에 근거하여 유전정보 인지군과 미인지군의 피험자 수의 비율을 2:1로 함.

2) 실험군과 대조군의 baseline characteristic에서 차이가 심할 경우 결과 해석에 어려움이 있을 것으로 예상되기 때문에 무작위 배정이 아닌 매치 배정을 통해 연구 참여자를 배정함. 연구 참여자의 성별과 체중, 나이를 매치하여 실험군과 대조군의 특성에 의한 지표의 차이를 최소화함.

**6. 연구 디자인**

**6.1 연구참여자 모집과정의 진행**

IRB에서 승인받은 모집공고문을 이용하여 오프라인 (교내 게시판 부착하여 공고) 및 온 라인 (교내 포탈 사이트 메일 및 자유게시판에 공고)으로 홍보하며, 이를 통해 자발적으로 담당자에게 직접 연락을 취해 온 대상에 대해서만 모집을 실시함. 모집 조건에 해당되는 연구 참여 대상은 25-35세의 성인으로 BMI (kg/㎡) 18.5 이상 25 미만이며, 제외 대상 질병이 없는 사람임.

**6.2 연구 동의과정의 진행**

IRB로부터 승인받은 동의서를 이용하여 동의 과정을 진행하며, 연구 진행 전에 따로 일시를 잡아 연구담당자가 연구에 대한 자세한 설명과 함께 동의서에 대한 내용을 적절히 설명하고, 이를 참가자들이 스스로 모두 숙지하도록 한 후, 최종적으로 참가자들의 스스로의 판단 하에 참여하고자 하는 의향이 변동이 없는 경우 동의서에 서명을 받게 됨. 단, 연구책임자의 실험실 소속 대학원 학생이나 강의 수강생은 배제함.

**6.3 실제적인 연구수행 과정**

**6.3.1 혈액수집 및 혈액검사**

혈액은 서울대 보건진료소의 채혈 전문가에게 의뢰하여 12시간 공복 정맥혈 회차 당 총 11 mL씩 총 3회를 채혈함. 혈액 중의 CBC, 빈혈지표, 공복혈당, 혈청 콜레스테롤, 중성지방, LDL 콜레스테롤, HDL 콜레스테롤, 간기능 지표를 측정함. Baseline, 3, 6개월 시점에서 각 회당 11 mL씩 채혈하여 검사를 진행함. 이 중 5 mL는 CBC, 빈혈지표, 공복혈당, 혈청 총콜레스테롤, 중성지방, LDL 콜레스테롤, HDL 콜레스테롤, 간기능 지표 등 임상지표 분석을 위해 사용. 3mL는 EDTA tube에 수집하여 유전자분석을 위해 사용, 3mL는 SST tube에 채혈하여 대사체 분석 및 추가 분석을 위해 이용함.

**6.3.2 체격지수 측정**

체격지수는 서울대학교 식품영양학과 연구실에서 측정함. InBody720을 이용하여 체중, 근육량, 지방량은 측정하고, 신장, 허리둘레를 측정하여 BMI와 복부비만도를 측정함. 체격지수 측정은 baseline, 1, 2, 3, 6 개월 시점에서 실시함.

**6.3.3 DTC 유전자 검사**

본 연구의 목적인 유전자 검사결과의 인지가 소비자의 건강관련 행동변화에 미치는 영 향을 알아보는 것과 동시에 식사섭취, 미각, 대사체 등에 영향을 미치는 대표성 있는 유 전자를 발굴하기 위해 baseline 시점에서 채혈한 혈액으로 본 연구의 연구 담당자가 유 전자체분석 전문기업으로서 유전체 서비스 및 개인 유전자분석 서비스 등을 제공하는 검사 전문 업체인 “테라젠이텍스”에 유전자 분석을 의뢰하며, Theragen PMRA chip을 이용하여 Affymetrix Axiom Custom Assay Plate protocol에 따라 분석함. 본 연구에서 사용하는 Theragen Precision Medicine Research Array (PMRA) chip은 ThermoFisher에서 개발한 Axiom 기반의 Asian PMRA chip의 약 75만 개 SNP를 기반으로 하여 테라젠이 연구와 서비스를 통해 축적한 5만 개의 SNP를 추가한 chip으로서, 임상적인 진단용 SNP들과 한국인 희귀빈도 변이 등을 추가해서 다양한 연구목적에 적합한 칩임. 유전자 검사 결과 연구 참여자에게는 보건복지부 고시 (제2016-97호)로 정해져 있는 총12가지 항목 (체질량지수, 중성지방농도, 콜레스테롤, 혈당, 혈압, 색소 침착, 탈모, 모발 굵기, 피부 노화, 피부 탄력, 비타민 C 농도, 카페인 대사)에 해당되는 유전정보를 알려줄 것이며, 위의 12개 항목에 대한 유전자는 모두 Theragen PMRA chip 분석에 포함되어 있음.

**6.3.4 대사체 분석**

대사체는 serum 50 µL를 이용하여 GC-MS/MS 방법으로 서울대학교 농업생명과학대학 농생명공학부 농약화학 및 독성학 연구실에서 분석함.

Methoximation/trimethylsilylation유도체화를 한 후 monosaccharide, 아미노산, 지방산, 핵산, 콜레스테롤, sugar alcohol, 대사 중간산물 (TCA cycle, glycolysis, urea cycle 등), 비타민 (비오틴, 비타민 C) 등 340 가지 지표를 측정함. Baseline, 3, 6개월 시점에서 측정함.

**6.3.5 맛에 대한 민감도 측정**

단맛과 짠맛에 대한 민감도를 측정하기 위해 설탕 (sugar)과 소금 (sodium chloride)을 이 용하여 농도가 증가하는 순으로 제공하는데, 중간에 맹물을 제공하면서 signal의 맛을 느 끼는 농도를 3-AFC 설문지에 기입하여 분석함. Baseline 시점에서 측정함.

**6.3.6 식습관 및 영양소 섭취 조사**

식품섭취빈도조사지 (FFQ, 국민건강영양조사 식품섭취빈도조사표)를 이용하여 지난 1년 간 또는 3개월 간의 식이 섭취 실태를 파악하고, 주중 2일과 주말 1일의 식사 일지를 작성하여 에너지와 영양소 섭취 실태를 분석함. FFQ를 사용한 분석은 baseline, 3, 6개월 시점에서 실시하고, 식사일지는 baseline, 1, 2, 3, 6 개월 시점에서 작성하여 추적 조사함.

**6.3.7 심리적 요인 검사**

우울/불안 (PHQ-9, GAD-7), 건강관련 삶의 질 (SF-36), 자아존중감 (Rosenberg의 자아존중감 척도), 충동성 (BIS-11) 영역의 측정을 위해 각 영역에 해당하는 설문지를 이용해 웹기반 설문조사 사이트인 survey monkey (https://ko.surveymonkey.com)를 통해 온라인으로 실시함. 또한, 충동성의 추가적인 측정을 위해서 충동성과 관련된 만족지연 능 력을 측정하는 지연 디스카운팅 (delay discounting) 과제를 함.

**6.3.8 신체활동량 측정**

웨어러블 디바이스 (Fitbit charge2)를 이용하여 활동량 (이동거리, 걸음수, 소모칼로리, 활동시간), 수면량 변화 등을 baseline, 1, 2, 3, 6개월 시점에서 10일간의 활동에 대해 측 정함. 또한, 각 시점에서 운동의 종류와 활동량을 연결하기 위해 운동의 강도에 따라 질 문을 분류한 신체활동 관련 설문인 신체활동 관련 설문인 IPAQ (International Physical Activity Questionnaire)-short form의 문항을 이용하여 웹 기반 설문조사 사이트인 survey monkey (https://ko.surveymonkey.com)를 통해 온라인으로 설문 조사를 실시함

**6.4 설문지에서 질문하는 내용**

**6.4.1 참가자의 기초 인적정보 관련**

본 연구에서는 연구 참여자들의 개인정보 노출을 최대한 피하기 위해 연구 시작 후 임 의의 피험자 번호를 배정하여 연구를 진행하므로, 연구에서 사용하는 모든 설문조사 에는 피험자 번호 외의 인적정보가 필요치 않음 (다만, 피험자에게 연락을 하기 위해서 연락 담당자는 성명과 연락처를 알아야 함). 피험자 번호는 바코드를 활용하여 관리하며, 피험자의 개인정보와 피험자번호의 매치는 피험자와 직접적인 대면을 하지 않는 1인만 알고 있도록 관리함.

**6.4.2 설문의 종류와 내용**

• 심리적 요인 관련 설문

- PHQ-9: 주요우울장애 (Major depressive disorder)의 가능성에 대한 평가로서 9문항으로 구성됨.

- GAD-7: 범불안장애 (Generalized Anxiety Disorder)의 가능성에 대한 평가로 7문항으로 구성됨.

- SF-36: 건강관련 삶의 질에 대한 설문으로서 신체적/정신적 상태와 그로 인한 일상 활 동에 미치는 영향에 대한 질문으로 36가지 문항으로 구성됨.

- Rosenberg의 자아존중감 척도: 자기 자신에 대해 얼마나 가치를 부여하는가에 대한 10 가지 문항으로 구성됨.

- BIS-11: 충동성 측정을 위한 평가로서 16가지 문항으로 구성됨.

• 맛에 대한 민감도 측정 관련 평가지

- 3-AFC: 각 회차에서 제공된 3가지 자극 중 다르게 느껴지는 한 가지를 찾아내어 표시함

• 신체활동 관련 설문

- IPAQ-SF: 운동 강도에 따른 활동 시간 및 빈도를 평가하는 7가지 문항으로 구성됨.

**7. 혈액 및 혈액을 이용한 실험기구의 폐기**

IRB에서 제공한 인체유래물 관리대장 법정서식을 이용하여 연구를 수행하며, 혈액 및 혈 액을 이용한 실험기구의 폐기는 biohazard waste 처리 규정에 의거하여 분리 폐기하며, 시료 및 검체를 확인할 수 없도록 아이디를 제거한 뒤 폐기함. 서울대학교 보건 진료소 임상검사실에서 혈액지표의 측정을 위하여 사용한 혈액 샘플의 폐기는 검사대상물폐기 대장에 기록함.

**8. 통계 분석**

각 지표들의 결과는 그 결과가 정상분포를 이루는 지 분포도를 조사하며 정상분포를 이루지 않을 경우 적절한 transformation을 함. 각 시험대상자의 지표 변화는 repeated measure임을 고려하여 통계처리 함. 실험군과 대조군의 전 후 비교는 paired t-test를 이 용하며 비교하며, 실험군과 대조군의 차이는 변화에 대한 비교를 independent t-test로검 증함.

**9. 연구참여자에 대한 안전성의 배려**

본 연구를 위해 실시되는 검사의 결과는 "DTC 유전자검사 결과의 인지가 심리적 영역과 식생활 및 건강관련 행동에 미치는 영향”을 알아보는 목적 외에는 이용되지 않음. 채혈은 학내 보건진료소에서 채혈전문가의 도움을 받아 채혈을 진행하며, 채혈 전 연구 참여자 에게 채혈 과정 중에 생길 수 있는 부작용인 통증, 타박상 및 쇼크로 인한 현기증이 발생할 수 있음을 설명함. 만약 이러한 부작용이 발생하면 즉시 응급처치 (지혈, 냉찜질 등) 등의 필요한 조치를 하고, 치료과정에 대한 모든 책임을 연구 책임자 및 담당자가 짐. 인체 시료를 안전하게 사용하기 위한 연구 참여자의 안전교육을 실시함. 연구 참여자가 본 연구에 참여 전 또는 참여 중 또는 그 이후에 언제든지 참여 중단 의사를 밝히는 경우에는 연구자는 참여를 그만둘 수 있도록 배려함. 연구자는 참여 중단 의사를 밝힌 대상자에게 필요한 경우 적절한 조치를 취하기 위하여 중단 이유를 물어볼 수는 있으나 질문에 대해 연구참여자는 스스로의 판단에 따라 묵비권을 행사할 수 있으며, 연구자는 이를 존중함. 연구 참여자가 본 연구에 계속 참여하기를 원치 않을 경우 이에 따른 연구 참여자에 대한 어떠한 불이익도 없을 것임. 중도 포기 시, 연구 참여자의 성별, 나이 등 비 식별정보 (인구 통계학 정보)와 baseline에서 수집한 정보는 분석에 사용할 수 있으므로 보관하며, 개인 정보 (이름, 연락처 등)는 모두 즉시 폐기할 것이며, 개인정보가 삭제된 데이터는 사용됨.

**10. 연구 수행 일정**

총 연구기간 2018.08.01~2019.07.31

(1) 2018년 8월 ~ 2018년 11월

- 생명윤리위원회 승인 신청, 연구방법 검토 및 확립

(2) 2018년 12월~ 2019년 5월

- 0, 1, 2, 3, 6 개월에 In body 및 체격지수측정, 신체활동 측정, 식사 섭취량 측정

- 0, 3, 6개월에 신체활동 측정, 식사 섭취량 측정, 심리적 요인 분석, 체격지수 측정, 임상 지표 분석, 대사체 분석

(3) 2019년 6월~ 2019년 7월

- 연구결과 정리, 보고서 및 논문 작성

**11. 연구의 윤리적 수행을 위해 필요한 사항**

각 참여자가 연구 참여 신청 시 참여자에게 연구 일정 공지, 사례비 지급 및 조사 결과 구 분을 위해 성명, 성별, 생년월일, 연락처, 계좌번호를 수집하는 단계가 있음. 수집한 개인 정보는 참여자의 권익이 최대한 존중될 수 있도록 특별한 주의를 기울여 연구책임자만 접근할 수 있는 곳에 보관함. 연구 참여 6개월 시점에 방문한 후 마지막으로 할당된 사례비를 지급할 예정이며, 연구 수행 기간으로 설정된 1년 동안 보관하여 연구 참여자들에게 사례비 지급 유무에 대한 확인 또는 불만사항, 또는 실험 참여에 관한 애로사항 등에 대한 불만 접수 등이 들어오지 않을 경우 서류 분쇄기를 이용하여 폐기처분 하고자 함. 전자적 파일은 복원이 불가능한 방법으로 영구 삭제함. 연구 진행 시 연구 담당자는 모든 연구 참여자에게 임의로 배정된 코드번호와 barcode system을 이용하여 연구를 수행함. 연구 참여자에게 제공되는 모든 온라인과 문서 형태의 설문지, 식사기록지, 식품섭취빈도조사지 등에도 barcode 및 코드 번호를 기입하여 피험자의 인적사항 및 기타 사항의 노출이 최소화되도록 함. 비밀 보장을 해 관련규정이 정하는 범위 안에서만 수행하며, 연구 결과 분석 외에 다른 용도로 공개되지 게 함. 또한, 만약 이 연구에서 얻어진 개인 정보가 학회지나 학회에 공개될 때는 연구 참여자의 이름을 비롯한 다른 개인정보는 사용하지 않을 것임을 연구 참여자에게 충분히 설명함. 연구 동의서는 생명윤리 법에 근거하여 연구 종료 후 3년이 지난 시점에서 파기하며, 연구 자료는 본교 연구윤리 지침에 근거하여 가능한 영구 보관할 수 있는데, 추후 논문 작성이나 관련 주제로 심화되어 연구할 경우 추가 분석이 필요할 수 있으므로 이를 고려하여 보관함.

연구 참여자를 대상으로 IRB에서 승인받은 동의서를 이용하여 동의 과정을 진행함. 헬 싱키 선언에 입각하여 연구 진행 전에 참여자들에게 미리 연구에 대한 충분한 설명을 하기 위해 연구 진행에 관한 오리엔테이션을 실시하여, 연구담당자가 연구에 대한 자세한 설명과 함께 동의서에 대한 내용을 적절히 설명하고, 이를 참가자들이 스스로 모두 숙지하도록 함. 최종적으로 스스로 판단 하에 참여하고자 하는 의향에 변동이 없는 경우 동의서에 자발적으로 서명을 받게 됨.

수집된 채혈은 향후 혈액검사, 유전자 검사 및 대사체 분석에 이용되며 그 결과는 연구 참여자가 원하는 경우에 한하여 본인에게만 알려줌. 또한, "생명윤리 및 안전에 관한 법률" 제39조 1항 “인체유래물연구자는 인체유래물 연구 동의서의 법정 서식”에 기재한 기간을 적용하여 보관 기간 종료 후 바로 폐기함. 혈액은 222동 622호 임상 및 면역 영 양학 실험실의 초저온 냉동고에 보관하며, 보관 장부를 두어 시료의 관리, 추적함. 혈 액의 사용, 보관 및 폐기는 연구 책임자와 연구 담당자가 책임을 짐.

**11. 참고문헌**

Hollands GJ, French DP, Griffin SJ, Prevost AT, Sutton S, King S et al. The impact of communicating genetic risks of disease on risk-reducing health behaviour: systematic review with meta-analysis. BMJ. 2016;352:i1102

Dias AG, Rousseau D, Duizer L, Cockburn M, Chiu W, Nielsen D et al. Genetic variation in putative salt taste receptors and salt taste perception in humans. Chem Senses. 2013;38(2):137-45

Dias AG, Eny KM, Cockburn M, Chiu W, Nielsen DE, Duizer L et al. Variation in the TAS1R2 Gene, Sweet Taste Perception and Intake of Sugars. J Nutrigenet Nutrigenomics. 2015;8(2):81-90

Sorkin R, Wolever T, El-Sohemy A. Genetic variation in the AMY1 gene is associated with dietary carbohydrate and starch intake in a young adult population. FASEB J 2017;31:S299.5

Torres SJ, Nowson CA. Relationship between stress, eating behavior, and obesity. Nutrition. 2007 (11-12):887-94

Hodge A, Almeida OP, English DR, Giles GG, Flicker L. Patterns of dietary intake and psychological distress in older Australians: benefits not just from a Mediterranean diet. Int Psychogeriatr. 2013;25(3):456-66

Lunn TE, Nowson CA, Worsley A, Torres SJ. Does personality affect dietary intake? Nutrition. 2014;30(4):403-9

Chol BY and Chung KM. Utility of Delay Discounting Task as a Measure of Impulsivity. Kor J Psychol. 30(4), 2011.11

Stewart-Knox BJ, Simpson EE, Parr H, Rae G, Polito A, Intorre F et al. Zinc status and taste acuity in older Europeans: the ZENITH study. Eur J Clin Nutr. 2005;59 Suppl 2:S31-6

Nielsen DE and EL-Sohemy A. Disclosure of Genetic Information and Change in Dietary Intake: A Randomized Controlled Trial. PLoS One. 2014 Nov 14;9(11)
