# Supplementary material for: Changes in anxiety and depression levels and meat intake following recognition of low genetic risk for high body mass index, triglycerides, and lipoproteins: A randomized controlled trial
Source: PLoS One. 2023 Sep 8;18(9):e0291052. doi: 10.1371/journal.pone.0291052 (PMC10490956; doi:10.1371/journal.pone.0291052)
Supplement: S3 File — (DOCX) [file pone.0291052.s014.docx]

**P1 Table. Differences in anthropometric measures among CON, ILR, and IHR groups ^1), 2), 3)^**

| **Total subjects (*n* = 100)** | | | | | | | |
| --- | --- | --- | --- | --- | --- | --- | --- |
| **Parameter** | **CON**  **(*n* = 32)** | | **ILR**  **(*n* = 28)** | | **IHR**  **(*n* = 31)** | | ***P* value ^2)^** |
|  | **Mean (SEM)** | **Change ^1)^** | **Mean (SEM)** | **Change ^1)^** | **Mean (SEM)** | **Change ^1)^** |  |
| **BMI (kg/m^2^)** | | | | | | | |
| Baseline | 22.4 (0.3) |  | 21.7 (0.4) |  | 22.7 (0.3) |  |  |
| 3-month follow-up | 22.2 (0.4) ***** | -0.1 (0.1) | 21.6 (0.4) ***** | -0.3 (0.1) | 22.5 (0.4) | -0.2 (0.1) | 0.668 |
| 6-month follow-up | 22.1 (0.4) | -0.2 (0.1) | 21.6 (0.4) | -0.1 (0.1) | 22.6 (0.4) | -0.1 (0.2) | 0.381 |
| **Body fat mass (kg)** | | | | | | | |
| Baseline | 16.3 (0.6) |  | 14.8 (0.6) |  | 15.9 (0.7) |  |  |
| 3-month follow-up | 15.2 (0.5) ***** | -0.8 (0.3) | 13.4 (0.8) ***** | -1.3 (0.3) | 14.7 (0.7) ***** | -1.1 (0.3) | 0.470 |
| 6-month follow-up | 15.0 (0.5) ***** | -0.8 (0.4) | 13.8 (0.7) ***** | -0.8 (0.3) | 15.5 (0.7) | -0.6 (0.3) | 0.391 |
| **Waist-to-hip ratio (%)** | | | | | | | |
| Baseline | 0.86 (0.01) |  | 0.85 (0.01) |  | 0.85 (0.01) |  |  |
| 3-month follow-up | 0.84 (0.01) ***** | -0.02 (0.00) | 0.83 (0.01) ***** | -0.02 (0.00) | 0.83 (0.01) ***** | -0.02 (0.00) | 0.153 |
| 6-month follow-up | 0.84 (0.01) ***** | -0.02 (0.00) **^a^** | 0.83 (0.01) ***** | -0.01 (0.00) **^ab^** | 0.84 (0.01) | -0.01 (0.00) **^b^** | 0.050 |
| **Skeletal muscle mass (kg)** | | | | | | | |
| Baseline | 26.4 (1.0) |  | 26.7 (1.2) |  | 26.9 (1.1) |  |  |
| 3-month follow-up | 26.6 (1.1) ***** | 0.2 (0.2) | 27.5 (1.2) ***** | 0.3 (0.1) | 27.1 (1.1) ***** | 0.2 (0.2) | 0.817 |
| 6-month follow-up | 26.5 (1.2) ***** | 0.2 (0.2) | 27.1 (1.2) | 0.2 (0.1) | 26.4 (1.1) | 0.1 (0.2) | 0.768 |

**^1)^** Change = anthropometric measurements at follow-up – anthropometric measurements at baseline.

**^2)^** Means with different superscripts indicate the significant differences in changes in anthropometric measurements among CON, INR, and IR groups by one-way ANOVA tests and Kruskal-Wallis tests, followed by Bonferroni-correction multiple comparison tests.

The asterisks indicate a significant difference (*: P value < 0.05) in the anthropometric measurements from the baseline to the follow-up time point.

**^3)^** Added sugar includes sugar, fruit juice, and sugar-sweetened beverages.

CON, control; ILR, Intervention-Low Risk; Intervention-High Risk

**P2 Table. Differences in nutrients and food intakes among CON, ILR, and IHR groups ^1), 2), 3)^**

| **Total subjects (*n* = 100)** | | | | | | | |
| --- | --- | --- | --- | --- | --- | --- | --- |
| **Parameter** | **CON**  **(*n* = 33)** | | **ILR**  **(*n* = 28)** | | **IHR**  **(*n* = 31)** | | ***P* value ^2)^** |
|  | **Mean (SEM)** | **Change ^1)^** | **Mean (SEM)** | **Change ^1)^** | **Mean (SEM)** | **Change ^1)^** |  |
| **Energy (kcal)** | | | | | | | |
| Baseline | 1934.8 (72.0) |  | 1809.2 (85.0) |  | 1916.5 (97.6) |  |  |
| 3-month follow-up | 1682.0 (66.3) ***** | -249.0 (71.8) | 1691.1 (99.4) | -135.1 (116.2) | 1716.4 (81.0) | -200.2 (92.7) | 0.694 |
| 6-month follow-up | 1806.6 (69.1) | -119.4 (74.2) | 1690.1 (102.4) | -135.3 (137.8) | 1759.7 (89.9) | -142.9 (109.6) | 0.987 |
| **Carbohydrate (% energy/day)** | | | | | | | |
| Baseline | 49.2 (1.1) |  | 48.9 (1.4) |  | 45.2 (1.5) |  |  |
| 3-month follow-up | 55.4 (1.8) ***** | 5.8 (2.1) **^a^** | 47.4 (2.2) | -1.5 (2.5) **^b^** | 49.4 (2.4) | 4.2 (2.7) **^ab^** | 0.021 |
| 6-month follow-up | 49.2 (1.4) | -0.2 (1.5) | 50.2 (1.8) | 1.4 (2.1) | 47.6 (1.7) | 2.3 (2.0) | 0.595 |
| **Protein (% energy/day)** | | | | | | | |
| Baseline | 15.8 (0.5) |  | 16.1 (0.6) |  | 17.0 (0.8) |  |  |
| 3-month follow-up | 16.2 (0.7) | 0.7 (0.7) **^ab^** | 19.2 (1.0) ***** | 3.1 (1.0) **^a^** | 17.2 (0.8) | 0.1 (1.1) **^b^** | 0.031 |
| 6-month follow-up | 16.6 (0.4) | 1.1 (0.6) | 16.1 (0.7) | -0.0 (1.1) | 17.3 (0.5) | 0.2 (0.8) | 0.636 |
| **Fat (% energy/day)** | | | | | | | |
| Baseline | 32.5 (0.9) |  | 32.7 (1.1) |  | 32.2 (1.1) |  |  |
| 3-month follow-up | 31.6 (1.6) | -0.8 (1.6) | 35.7 (1.4) | 3.0 (1.9) | 34.7 (1.9) | 2.5 (1.9) | 0.046 |
| 6-month follow-up | 33.1 (1.2) | 0.5 (1.0) | 32.2 (1.5) | -0.6 (1.8) | 33.7 (1.5) | 1.5 (1.8) | 0.625 |
| **Meat (g/day)** | | | | | | | |
| Baseline | 105.0 (12.5) |  | 94.0 (12.2) |  | 140.2 (16.0) |  |  |
| 3-month follow-up | 99.6 (14.5) | -3.9 (18.5) **^ab^** | 139.6 (18.2) ***** | 45.8 (21.1) **^a^** | 105.5 (10.0) | -34.7 (19.4) **^b^** | 0.027 |
| 6-month follow-up | 113.4 (14.5) | 9.8 (18.2) | 106.5 (19.3) | 10.3 (22.4) | 133.0 (15.8) | -8.5 (18.5) | 0.997 |
| **Fruits (g/day)** | | | | | | | |
| Baseline | 86.6 (15.6) |  | 72.4 (17.3) |  | 112.2 (19.2) |  |  |
| 3-month follow-up | 71.2 (15.9) | -16.8 (18.8) **^ab^** | 83.8 (23.9) | 8.1 (28.6) **^a^** | 48.8 (10.3) ***** | -68.2 (18.6) **^b^** | 0.038 |
| 6-month follow-up | 71.0 (11.5) | -17.0 (18.0) | 62.7 (17.1) | -15.7 (15.1) | 66.9 (20.2) | -44.6 (26.2) | 0.593 |
| **Added sugar (g/day)** | | | | | | | |
| Baseline | 125.3 (24.6) |  | 60.5 (14.5) |  | 62.5 (15.0) |  |  |
| 3-month follow-up | 71.6 (14.9) ***** | -57.2 (28.2) | 61.5 (14.9) | 7.1 (21.0) | 78.9 (20.0) | 14.2 (26.8) | 0.119 |
| 6-month follow-up | 73.0 (18.4) ***** | -55.7 (26.9) **^b^** | 74.5 (16.6) | 18.1 (23.8) **^ab^** | 113.5 (26.4) ***** | 47.4 (22.1) **^a^** | 0.007 |

**^1)^** Change = nutrients or food intake at follow-up – nutrients or food intake at baseline.

**^2)^** Means with different superscripts indicate the significant differences in changes in nutrients or food intake among CON, INR, and IR groups by one-way ANOVA tests and Kruskal-Wallis tests, followed by Bonferroni-correction multiple comparison tests.

The asterisks indicate a significant difference (*: P value < 0.05) in the nutrients or food intake from the baseline to the follow-up time point.

**^3)^** Added sugar includes sugar, fruit juice, and sugar-sweetened beverages.

CON, control; ILR, Intervention-Low Risk; Intervention-High Risk

**P3 Table. Differences in anxiety and depression symptom levels among CON, ILR, and IHR groups ^1), 2)^**

| **Total subjects (*n*=91)** | | | | | | | |
| --- | --- | --- | --- | --- | --- | --- | --- |
| **Parameter** | **CON**  **(*n* = 32)** | | **ILR**  **(*n* = 28)** | | **IHR**  **(*n* = 31)** | | ***P* value ^2)^** |
|  | **Mean (SEM)** | **Change ^1)^** | **Mean (SEM)** | **Change ^1)^** | **Mean (SEM)** | **Change ^1)^** |  |
| **GAD-7_Score** | | | | | | | |
| Baseline | 3.2 (0.6) |  | 4.3 (0.8) |  | 3.5 (0.5) |  |  |
| 3-month follow-up | 3.5 (0.8) | 0.3 (0.7) | 3.6 (0.9) | -0.9 (1.0) | 3.5 (0.6) | 0.0 (0.7) | 0.833 |
| 6-month follow-up | 4.1 (0.9) | 0.7 (0.9) | 3.1 (0.8) | -1.3 (0.9) | 3.3 (0.5) | -0.1 (0.7) | 0.968 |
| **PHQ-9_Score** | | | | | | | |
| Baseline | 4.5 (0.8) |  | 6.8 (0.9) |  | 4.2 (0.5) |  |  |
| 3-month follow-up | 4.9 (0.8) | 0.5 (0.7) | 5.3 (0.9) | -1.5 (0.9) | 4.8 (0.7) | 0.5 (0.7) | 0.217 |
| 6-month follow-up | 5.7 (1.1) | 1.1 (1.2) **^a^** | 3.8 (0.8) ***** | -2.9 (0.9) **^b^** | 4.4 (0.6) | 0.1 (0.7) **^ab^** | 0.027 |
| **Male subjects (n=45)** | | | | | | | |
| **Parameter** | **CON**  **(*n* =16)** | | **ILR**  **(*n* =14)** | | **IHR**  **(*n* =15)** | | ***P* value ^2)^** |
|  | **Mean (SEM)** | **Change ^1)^** | **Mean (SEM)** | **Change ^1)^** | **Mean (SEM)** | **Change ^1)^** |  |
| **GAD-7_Score** | | | | | | | |
| Baseline | 3.9 (0.8) |  | 3.8 (1.2) |  | 3.4 (0.7) |  |  |
| 3-month follow-up | 4.6 (1.2) | 0.6 (1.1) | 5.7 (1.5) ***** | 1.6 (1.1) | 3.7 (0.7) | 0.3 (0.8) | 0.272 |
| 6-month follow-up | 3.6 (1.0) | -0.4 (0.9) | 4.4 (1.4) | 0.1 (0.7) | 3.4 (0.8) | -0.1 (1.0) | 0.496 |
| **PHQ-9_Score** | | | | | | | |
| Baseline | 5.6 (1.4) |  | 5.9 (1.5) |  | 4.6 (0.7) |  |  |
| 3-month follow-up | 6.2 (1.3) | 0.6 (1.3) | 7.4 (1.6) ***** | 1.1 (1.1) | 4.7 (0.8) | 0.1 (0.9) | 0.003 |
| 6-month follow-up | 4.8 (0.9) | -0.6 (1.2) | 4.7 (1.3) ***** | -1.1 (1.0) | 4.1 (0.8) | -0.6 (0.9) | 0.007 |
| **Female subjects (n=46)** | | | | | | | |
| **Parameter** | **CON**  **(*n* =16)** | | **ILR**  **(*n* =14)** | | **IHR**  **(*n* =16)** | | ***P* value ^2)^** |
|  | **Mean (SEM)** | **Change ^1)^** | **Mean (SEM)** | **Change ^1)^** | **Mean (SEM)** | **Change ^1)^** |  |
| **GAD-7_Score** | | | | | | | |
| Baseline | 3.9 (0.8) |  | 3.8 (1.2) |  | 3.4 (0.7) |  |  |
| 3-month follow-up | 4.6 (1.2) | 0.6 (1.1) | 5.7 (1.5) | 1.6 (1.1) | 3.7 (0.7) | 0.3 (0.8) | 0.639 |
| 6-month follow-up | 3.6 (1.0) | -0.4 (0.9) | 4.4 (1.4) | 0.1 (0.7) | 3.4 (0.8) | -0.1 (1.0) | 0.608 |
| **PHQ-9_Score** | | | | | | | |
| Baseline | 5.6 (1.4) |  | 5.9 (1.5) |  | 4.6 (0.7) |  |  |
| 3-month follow-up | 6.2 (1.3) | 0.6 (1.3) | 7.4 (1.6) | 1.1 (1.1) | 4.7 (0.8) | 0.1 (0.9) | 0.722 |
| 6-month follow-up | 4.8 (0.9) | -0.6 (1.2) | 4.7 (1.3) | -1.1 (1.0) | 4.1 (0.8) | -0.6 (0.9) | 0.944 |

**^1)^** Change = PHQ-9 or GAD-7 scores at follow-up – PHQ-9 or GAD-7 scores at baseline.

**^2)^** Means with different superscripts indicate the significant differences in changes in PHQ-9 and GAD-7 scores among CON, INR, and IR groups by one-way ANOVA tests and Kruskal-Wallis tests, followed by Bonferroni-correction multiple comparison tests.

The asterisks indicate a significant difference (*: P value < 0.05) in the PHQ-9 or GAD-7 scores from the baseline to the follow-up time point.

CON, control; ILR, Intervention-Low Risk; Intervention-High Risk

**P4 Table. Multiple linear regression analysis on the association between GAD-7 scores and meat intake in the ILR group ^1), 2)^**

| **Model** | **Variables** | **ILR group (n=28)** | | | | | | |
| --- | --- | --- | --- | --- | --- | --- | --- | --- |
|  |  | **GAD-7 score at 3-month** | | | **GAD-7 score at 6-month** | | | |
|  |  | **B (SE)** | **Standardized *β*** | ***p*** | **B (SE)** | **Standardized *β*** | ***p*** |  |
| **1** | **Meat intake (g/day)** | -0.018  (0.009) | -0.381 | 0.046 | -0.014  (0.009) | -0.292 | 0.166 |  |
| **2** | **Meat intake (g/day)** | -0.014  (0.008) | -0.292 | 0.085 | -0.014  (0.009) | -0.300 | 0.141 |  |
|  | **Women vs. men** | 3.738  (1.721) | 0.410 | 0.041 | 0.317  (1.735) | 0.041 | 0.857 |  |
|  | **Age (years)** | 0.860  (0.307) | 0.439 | 0.010 | 0.325  (0.399) | 0.167 | 0.426 |  |
|  | **BMI (kg/m^2^)** | -0.071  (0.412) | -0.032 | 0.865 | -0.804  (0.478) | -0.401 | 0.110 |  |
|  | **Total PA (MET-hrs/week)** **^1)^** | 0.031  (0.027) | 0.177 | 0.271 | -0.013  (0.029) | -0.091 | 0.651 |  |

**^1)^** MET: metabolic equivalent task

**^2)^** Model 1: R^2^ for GAD-7 scores at 3-month and 6-month were 0.145 and 0.085, respectively; Model 2: R^2^ for GAD-7 scores at 3-month and 6-month were 0.481 and 0.371, respectively.

ILR, Intervention-Low Risk; BMI, body mass index; PA, physical activity; GAD-7, Generalized Anxiety Disorder 7-item scale
