# Supplementary material for: Changes in anxiety and depression levels and meat intake following recognition of low genetic risk for high body mass index, triglycerides, and lipoproteins: A randomized controlled trial
Source: PLoS One. 2023 Sep 8;18(9):e0291052. doi: 10.1371/journal.pone.0291052 (PMC10490956; doi:10.1371/journal.pone.0291052)
Supplement: Attachment — Submitted filename: renamed_7a2a8.docx [file pone.0291052.s015.docx]

# COMMENTS – Manuscript PONE-D-22-19835

Title: “Changes in anxiety levels and meat intake following recognition of low genetic risk for high body mass index, triglycerides, and lipoproteins: A randomized controlled trial”

**Important note: This review pertains only to ‘statistical aspects’ of the study and so ‘clinical aspects’ [like medical importance, relevance of the study, ‘clinical significance and implication(s)’ of the whole study, etc.] are to be evaluated [should be assessed] separately/independently. Further please note that any ‘statistical review’ is generally done under the assumption that (such) study specific methodological [as well as execution] issues are perfectly taken care of by the investigator(s). This review is not an exception to that and so does not cover clinical aspects {however, seldom comments are made only if those issues are intimately / scientifically related & intermingle with ‘statistical aspects’ of the study}. Agreed that ‘statistical methods’ are used as just tools here, however, they are vital part of methodology [and so should be given due importance].**

**COMMENTS:** Your ABSTRACT is well drafted but assay type. Please note that it is preferable [refer to item 1b of CONSORT checklist 2010: Structured summary of trial design, methods, results, and conclusions] to divide the ABSTRACT with small sections like ‘Objective(s)’, ‘Methods’, ‘Results’, ‘Conclusions’, etc. which is an accepted practice of most of the good/standard journals [including this one, though ‘The PLoS One Guidelines to Authors’ did not specify an Abstract format, it is desirable]. It will definitely be more informative then, I guess, whatever the article type may be.

As you may know [it is well-known] that while reporting [findings from] ‘Clinical Trial’ one should follow CONSORT guidelines. Even important items {like How sample size was determined (Item 7a), Random Sequence generation (Item 8a), Allocation concealment (Item 9), Blinding (Item 11a)} of/in CONSORT checklist are not found [since your article type is ‘Clinical Trial’, you are supposed to cover these items in the report].

How the required minimum sample size for this study was determined is nevertheless a very-very important question for any type of study (clinical trial or else) which needs to be discussed in adequate details {including assumptions made at the time estimation, power of the study, software used, etc.}. Even how or why the allocation ratio of trial with a 2 (intervention):1 (control) subjects is decided is not given. When the allocation/assignment ratio used is different than 1:1, {which is definitely allowed but} one has to give the justification/reason (for this deviation) because then the ‘power’ [of comparison] is affected.

In section ‘5. Statistical analysis’ (line 173) it is said that “all analyses were performed using the intent-to-treat principle”. You may know well the importance & significance of ‘Per Protocol (PP)’ or Completers analyses but I request authors to refer/read section ‘5.2.3 Roles of the Different Analysis Sets’ discussed on page 24 of ‘ICH HARMONISED TRIPARTITE GUIDELINE - STATISTICAL PRINCIPLES FOR CLINICAL TRIALS, Publication-E9’.

It may also (please) be noted [in the backdrop of account given in section ‘5. Statistical analysis’, especially because you said “The differences in changes in nutrient and food intakes and psychological factors among the groups were analyzed using ANOVA for normally distributed data and Kruskal-Wallis tests for skewed data, followed by Bonferroni tests for multiple comparisons for post-hoc analysis. The changes in nutrient intakes and psychological factors were assessed using paired *t* tests for normally distributed data and Wilcoxon signed-rank tests for skewed data”] that for data that are in ‘ordinal’ level of measurement and not in ratio level of measurement for sure {as the score two times higher does not indicate presence of that parameter/phenomenon as double (for example, a Visual Analogue Scales VAS score or say ‘depression’ score)}. Then application of suitable non-parametric test(s) is/are indicated/advisable [even if distribution may be ‘Gaussian’ (also called ‘normal’)]. Agreed that there is/are no non-parametric test(s)/technique(s) available to be used as alternative in all situation(s) [suitable / most desired/applicable], but should be used whenever/wherever they are available.

Generally, all nutrient & food intakes and psychological factors data are in ‘ordinal’ level of measurement [definitely not in ratio level of measurement] and therefore here application of suitable non-parametric test(s) is/are indicated/advisable [even if distribution may be ‘Gaussian’ (also called ‘normal’)]. Moreover, note that ‘Multivariate linear regression’ needs continuous/ratio level data [as analyses were performed to determine the association between the psychological factors and dietary intakes].

It is appreciated that Spearman’s (and not Pearson’s) correlation coefficient was used [to assess the relationship between nutrients and meat intake] because as you know, Spearman’s correlation coefficient is non-parametric but Pearson’s correlation coefficient is parametric. However, remember that highly significant (large) value of ‘Pearson’s or Spearman’s correlation coefficient’ alone does not imply cause-effect relationship. There are other certain criteria which are to be considered before making any causal inference(s). Because ‘P-value’ heavily depends on sample size, it is customary to use the absolute value of ‘Correlation coefficient’ for interpreting positive or negative correlations (and do not rely only on corresponding ‘P’-value but also consider an absolute value of ‘Correlation coefficient’).

To provide a description of baseline characteristics is entirely reasonable (since it is clearly important in assessing to whom the results of the trial can be applied), however, statistical comparison of baseline characteristics [last (6^th^) ‘p-value’ column in Table 1] is not desirable at all [because even if P-value turns out to be significant (while comparing baseline characteristics despite random allocation), it is, by definition, a false positive] as you then are supposed to be testing ‘randomization’ then, which in any single trial may not balance all baseline characteristics [particularly when sample sizes are small] because ‘randomization’ is a sort of ‘insurance’ and not a guarantee scheme. Authors may please refer to following articles:

References:

1.	Stuart J. Pocock, et al., ‘Subgroup analysis, covariate adjustment and baseline comparisons in clinical trial reporting: current practice and problems’, Statistics in medicine, 2002; 21:2917–2930 [Particularly page 2927]

2.	Harrington D, et al., ‘New guidelines for statistical reporting in the journal’, N Engl J Med 2019;381:285-6

[Important message (indirectly/ultimately indicated) from these articles: Never do any comparison with respect to ‘baseline’ characteristics {by applying statistical significance test(s)}, when allocation is done randomly].

Good that (in lines 350-354) it is clarified that “This study has several limitations as well. Since the study sample was limited to healthy, young, well-educated adults, it might be questionable to generalize our findings to more general populations. Furthermore, although we observed significant changes in depression and anxiety levels among groups, the levels stayed within the normal range throughout the study period.” however, the last part [we observed significant changes in depression and anxiety levels among groups, the levels stayed within the normal range] is not clear. What exactly do you want/wish to indicate (by saying this)?
