# Supplementary material for: Changes in anxiety and depression levels and meat intake following recognition of low genetic risk for high body mass index, triglycerides, and lipoproteins: A randomized controlled trial
Source: PLoS One. 2023 Sep 8;18(9):e0291052. doi: 10.1371/journal.pone.0291052 (PMC10490956; doi:10.1371/journal.pone.0291052)
Supplement: Attachment — Submitted filename: Response to reviewers(PONE-D-22-19835R1).docx [file pone.0291052.s016.docx]

**Response to reviewers’ comments**

PONE-D-22-19835R1

Changes in anxiety and depression levels and meat intake following recognition of low genetic risk for high body mass index, triglycerides, and lipoproteins: A randomized controlled trial

PLOS ONE

Authors would like to thank the reviewers for kind explanations and valuable suggestions and comments that are necessary to improve this paper. Authors have acknowledged the concerns raised by reviewers and provided the response to reviewers’ comments and the revised version of the manuscript.

**Reviewer #1**

**COMMENTS: Since few [1] of the comments made on earlier draft [but frankly speaking, I am not very much convinced (or happy) for reasons given or arguments made {particularly the rationale for the allocation ratio of trial with 2 (intervention):1 (control)}, I feel, ‘let the respected editor decide the future course’. Note that ‘Response to Reviewers’ are not given separately as required [making it difficult read]. I do not have any specific recommendation [though only as system requirement, I choose minor revision].**

: Authors would like to apologize that the response was not clear enough and that the reviewer was not convinced with the rationale for the allocation. We have provided further explanation and reference for this. We applied a 2:1 ratio of subjects in the intervention group and the control group because the intervention group consisted of those with either “risk” or “no-risk” genotypes of genes in the genetic testing results [1]. This is provided as the rationale for the allocation ratio of trial with 2 (intervention): 1 (control) in the method part of the ‘Revised manuscript with track changes’ (p.8, lines 124-126).

1.	Nielsen DE, El-Sohemy A. Disclosure of genetic information and change in dietary intake: a randomized controlled trial. PLoS One. 2014;9(11):e112665. Epub 2014/11/15. doi: 10.1371/journal.pone.0112665. PubMed PMID: 25398084; PubMed Central PMCID: PMCPMC4232422 this manuscript has the following competing interests: AE-S holds shares in Nutrigenomix Inc., a genetic testing company for personalized nutrition. This does not alter the authors' adherence to PLOS ONE policies on data sharing and materials.

**Reviewer #2**

**I appreciate the authors thoughtful response to the previously offered comments. In some cases the comments were fully answered, and they have been removed from this response. In other cases I have offered an elaboration.**

**REVIEWERS RESPONSE TO AUTHORS ON POINT #2: There may be some miscommunication on this point. Given the topic that is being explored, it is crucial to contextualize it in the existing literature which has not generally indicated a strong psychosocial or behavioral response to DTC genetic results. This makes the findings of this study unusual, although not entirely inconsistent with occasional impacts that are observed. It is important to distinguish between responses from severe risks and minor risks as are conveyed in these DTC findings that were communicated to participants, and the literature review should be careful to not conflate the two. References #8 and 9 cited above are interesting, but largely obsolete, being over 15 years old. DTC testing did not become widely available until after 2007, which was after the publication of these papers. There has been widespread and extensive research on the topics examined by this scholarship that need to be acknowledged and integrated. For example, please see the review of 37 papers on the topic synthesized by Covolo et al, 2015.**

**Furthermore, for a review that explores a range of systematic reviews on this topic, consider examining Wade (2019).**

**Wade, Christopher H., “ What Is the Psychosocial Impact of Providing Genetic and Genomic Health Information to Individuals? An Overview of Systematic Reviews,” Looking for the Psychosocial Impacts of Genomic Information, special report, Hastings Center Report 49, no. 3 (2019): S88– S96. DOI: 10.1002/hast.1021**

**It is likely that there are more recent reviews which could be used; and the authors are encouraged to look for them. The context of this scholarship, and the way this study is situated within that field of study, should be developed further in both the introduction and the discussion sections.**

: Thank you very much for the detailed explanation and recommending reference of the recent review. The authors reviewed previous studies, focusing on the latest researches, and summarized the overall flow of the studies and the results according to the severity of the diseases in the discussion part of the ‘Revised manuscript with track changes’ (p.23-24, lines 384-396). Furthermore, we provided a revised introduction part reflecting the above point of view (p.5-6, lines 77-85). We have cited the more recent references in the revised manuscript.

**REVIEWERS RESPONSE TO AUTHORS ON POINT #3: This comment was more specifically focused on the measures used to evaluate psychological states. The measures used were standardized measures for global states, which tend to be insensitive to the mild/moderate responses typically observed for DTC genetic risk findings. Test-specific measures tend to perform better. To examine this issue further, please refer to the reviews mentioned above. It would be beneficial to comment on the measures used, the fact that they measure general states, and the implications for observing a significant difference in the discussion, particularly in light of the findings from other studies in this field.**

**GENERAL ADDED COMMENT: The authors may wish to add a concluding paragraph.**

: Authors acknowledged the need to comment on the measures we used and to discuss implications for the results of this study by mentioning the results of other studies that used condition-specific assessments. Therefore, we discussed this in the discussion part (p.24, lines 397-413) of the ‘Revised Manuscript with Track Changes’ and the modified introduction (p.5, lines 80-82) and conclusion (p.27, lines 477-481).
